# Supplementary material for: Real-time imaging reveals radiation-induced intratumor apoptosis via nutrient and oxygen deprivation following vascular damage
Source: Mol Ther Oncol. 2025 May 20;33(2):200997. doi: 10.1016/j.omton.2025.200997 (PMC12166797; doi:10.1016/j.omton.2025.200997)
Supplement: Document S1. Figures S1–S3 [file mmc1.pdf]

OMTON, Volume *33*

Supplemental information

Real-time imaging reveals radiation-induced intratumor apoptosis via nutrient and oxygen deprivation following vascular damage

Go Kagiya, Ryohei Ogawa, Toshihide Matsumoto, Fuminori Hyodo, Nanako Abe, Ami Yuzawa, Haru Takeuchi, Miki Aoyagi, Ayaka Sato, Kei Yamashita, and Masanori Hatashita

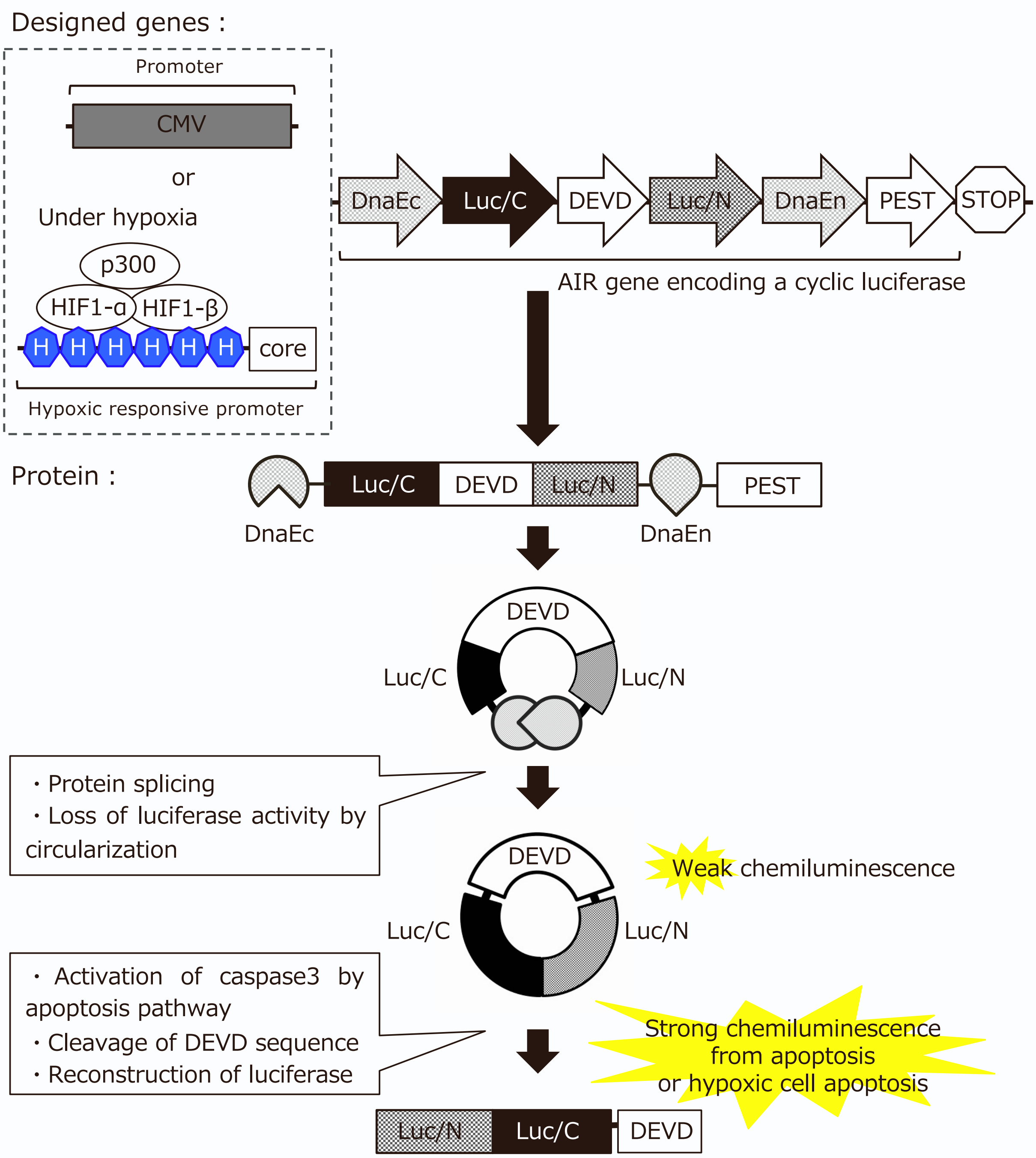

Figure S1. Schematic illustration for the detection of apoptosis and hypoxic cell apoptosis using apoptosis imaging reporter (AIR). In the plasmid used for detection of apoptosis, the expression of AIR is regulated by the CMV promoter. In contrast, for the detection of hypoxic cell apoptosis, a hypoxia-responsive promoter (HRP) that contains six hypoxia-responsive element and CMV IE1 core promoter, to which a heterodimeric complex composed of HIF-1α and HIF-1β binds, regulates the expression of AIR. Upon translation, the DnaEc and DnaEn inteins interact with each other to catalyze protein splicing, resulting in polypeptide circularization. If this event fails, rapid degradation occurs due to the PEST domain added at the N-terminus, which act as a signal peptide for protein degradation. The enzymatic activity of luc is lost when AIR distorted by circularization. However, the enzymatic activity is restored when the structure of luc becomes intact after caspase-3 cleaves the DEVD peptide in AIR during apoptosis. This results in high luminescence generation by luciferin oxidation.
CMV, human cytomegalovirus immediate early enhancer and promoter; H, hypoxia-responsive element; core, CMV IE1 core promoter; DnaEn, N-terminal fragment of DnaE intein; DnaEc, C-terminal fragment of DnaE intein; Luc/N, N-terminal fragment of luc; Luc/C, C-terminal fragment of luc; PEST, proteolytic signal to target proteins for degradation.

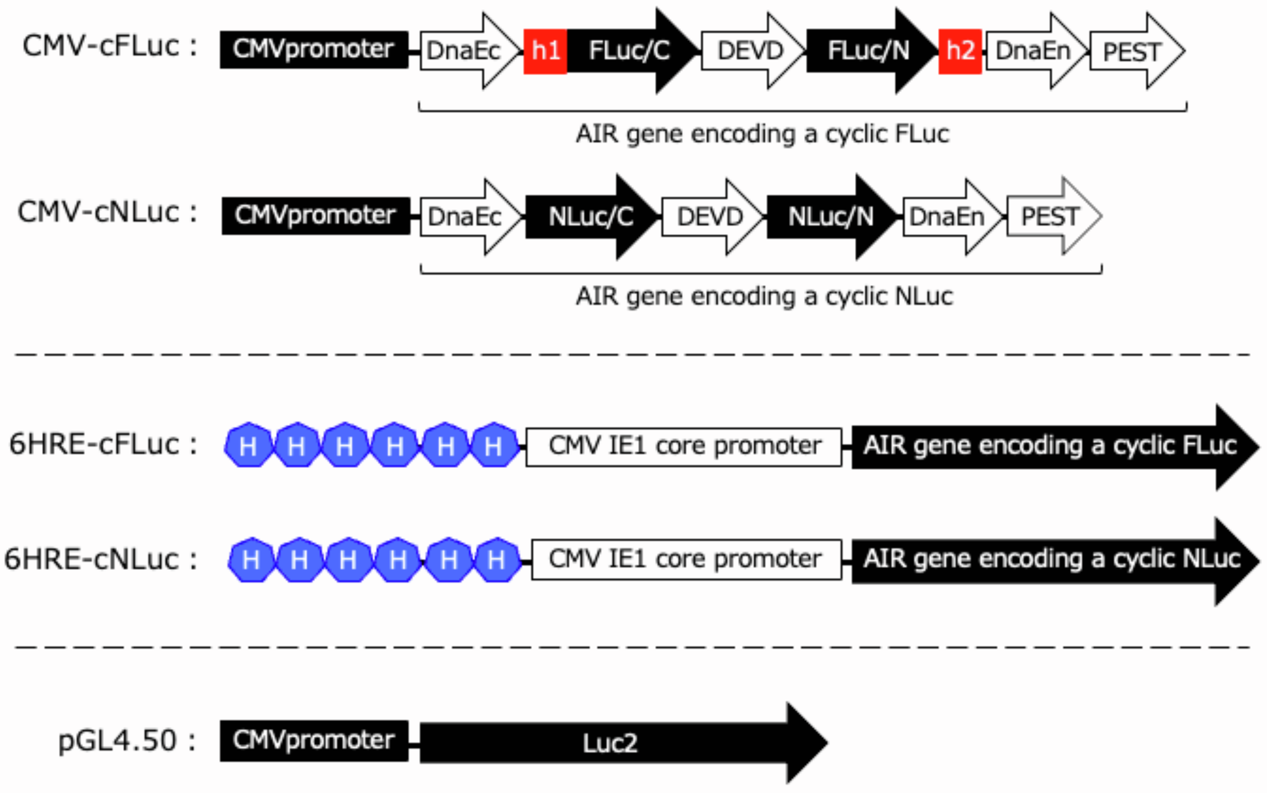

Figure S2. Schematic illustrations of the structures of the plasmids CMV-cFLuc, CMV-cNLuc, 6HRE-cFLuc, 6HRE-cNLuc, and pGL4.50 used in this study. The 'h1' and 'h2' in the top row represent two different hinge-array sequences. h1: CFNIS (one-letter amino acid sequence) and h2: KFAEYC (one-letter amino acid sequence). CMV promoter: CMV immediate early enhancer/promoter. The 'H' in the heptagon in the middle row denote the hypoxia-responsive element. H: cacgta (nucleotide sequence), Luc2: firefly luc gene.

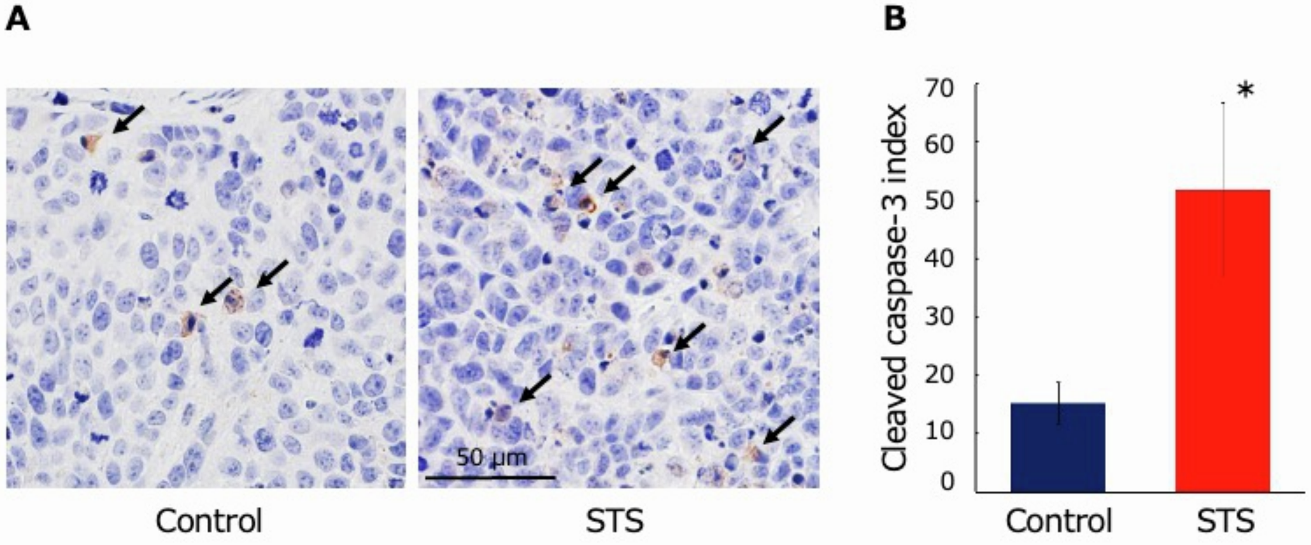

Figure S3. *In vivo* detection of STS-induced apoptotic cells using cleaved caspase-3 staining. A, Detection of cleaved caspase-3–positive cells in tumors. Cell nuclei were stained with Mayer's hematoxylin and cleaved caspase-3 positive cells (arrows) were identified as apoptotic cells. **B,** Cleaved caspase-3 positive cells in STS-treated tumors vs. untreated tumors. Asterisks indicate a statistically significant difference (unpaired t-test, p = 0.01). Data are presented as the mean ± SD (n = 4).
