# Supplementary material for: Real-time imaging reveals radiation-induced intratumor apoptosis via nutrient and oxygen deprivation following vascular damage
Source: Mol Ther Oncol. 2025 May 20;33(2):200997. doi: 10.1016/j.omton.2025.200997 (PMC12166797; doi:10.1016/j.omton.2025.200997)
Supplement: Document S2. Article plus supplemental information [file mmc2.pdf]

Molecular Therapy
Oncology
Original Article

Real-time imaging reveals radiation-induced intratumor apoptosis via nutrient and oxygen deprivation following vascular damage

Go Kagiya,[1,2,12] Ryohei Ogawa,[3,12] Toshihide Matsumoto,[1,2] Fuminori Hyodo,[4] Nanako Abe,[5] Ami Yuzawa,[6,7] Haru Takeuchi,[7] Miki Aoyagi,[8] Ayaka Sato,[9] Kei Yamashita,[10] and Masanori Hatashita[11]

[1]School of Allied Health Sciences, Kitasato University, 1-15-1 Kitasato, Minami-ku, Sagamihara, Kanagawa 252-0373, Japan; [2]Regenerative Medicine and Cell Design Research Facility, Kitasato University, 1-15-1 Kitasato, Minami-ku, Sagamihara, Kanagawa 252-0373, Japan; [3]Department of Radiology, Faculty of Medicine, Academic Assembly, University of Toyama, 2630 Sugitani, Toyama 930-0194, Japan; [4]Department of Pharmacology, Graduate School of Medicine Gifu University, 1-1 Yanagido, Gifu 501-1194, Japan; [5]The Kitasato University Graduate School of Medical Sciences, 1-15-1 Kitasato, Minami-ku, Sagamihara, Kanagawa 252-0373, Japan; [6]Graduate School of Human Health Sciences, Tokyo Metropolitan University, 7-2-10 Higashi-Ogu, Arakawa-ku, Tokyo 116-8551, Japan; [7]Department of Radiation, The Jikei University Hospital, 3-19-18 Nishishinbashi, Minato-ku, Tokyo 105-8471, Japan; [8]Radiological Center, NTT Medical Center Tokyo, 5-9-22 Higashigotanda, Shinagawa-ku, Tokyo 141-8625, Japan; [9]Department of Radiology, St. Marianna University School of Medicine, 2-16-1 Sugao, Miyamae, Kawasaki, Kanagawa 216-8511, Japan; [10]Department of Radiation Oncology, Chiba Cancer Center, 666-2 Nitona-chou, Chuo-ku, Chiba 260-8717, Japan; [11]Biotechnology Division, The Wakasa Wan Energy Research Center, 64-52-1 Nagatani, Tsuruga, Fukui 914-0192, Japan

Radiotherapy exerts significant effects on the tumor microenvironment. Radiation is known to induce direct tumor cell death through intrinsic factors (e.g., DNA repair activity and p53-mediated radiation sensitivity). It also induces indirect cell death by altering the tumor microenvironment. However, current knowledge is based on indirect evidence. We focused on apoptosis, a form of cell death, to elucidate the mechanisms underlying radiation-induced apoptosis in tumors. Using apoptosis-imaging cells and real-time imaging technology, we investigated the pathways involved in radiation-induced apoptosis in the tumor environment and found that the mechanism triggering early apoptosis following X-ray exposure involves minimal participation of the immune system. Furthermore, we demonstrated that apoptosis in tumors is an indirect form of cell death as well, primarily driven by radiation-induced damage to the tumor vasculature, which leads to reduced blood flow, resulting in nutrient and oxygen deprivation within cancerous tissue, which induces apoptosis. The presence of nutrient deprivation and hypoxia, mediated by tumor vascular damage, suggests the possibility of inducing not only apoptosis but also other forms of cell death (e.g., autophagic cell death and necrosis-like cell death) based on these mechanisms. These pathways provide valuable insights for the development of more effective radiotherapy strategies.

INTRODUCTION

At least 50% of patients with cancer undergo radiation therapy,[1,2] and in recent years, treatments utilizing X-rays as well as particle beams, including protons, neutrons, and heavy charged particles, have become increasingly prevalent.[3] However, the therapeutic effects of radiation within tumor tissues depend not only on the cellular responses to radiation at the tumor cell level but also on the complex interactions with the surrounding tissue and immune cells that constitute the tumor. Many aspects of these processes remain unresolved, and the precise mechanisms underlying their therapeutic efficacy are still not fully understood.

Traditionally, the primary mechanism is believed to be the induction of direct cell death via cytotoxic effects.[2,4] However, recent studies highlighted that radiation therapy can also modulate anticancer immunity, potentially diminishing its overall effectiveness. This dual nature of radiation (promoting and suppressing anticancer immunity) has drawn increasing attention for its complex role in the tumor microenvironment.[5] Furthermore, growing evidence suggests that the radiosensitivity of endothelial cells within the tumor vasculature significantly influences the overall response of the tumor to radiation.[6,7] Nevertheless, the direct cytotoxic effects of radiation remain central to its anticancer activity and crucial for determining therapeutic outcomes.

Radiation exposure triggers apoptosis,[8] a form of programmed cell death. Signaling pathways involved in radiation-induced apoptosis have been extensively studied *in vitro* using cell models. DNA damage and cell membrane damage are the two main pathways through which radiation can induce apoptosis. In the DNA damage pathway, activated ataxia telangiectasia mutated (ATM) protein stabilizes

Received 27 November 2024; accepted 15 May 2025;
https://doi.org/10.1016/j.omton.2025.200997.

[12]These authors contributed equally

Correspondence: Go Kagiya, PhD, School of Allied Health Sciences, Kitasato University, 1-15-1 Kitasato, Minami-ku, Sagamihara, Kanagawa 252-0373, Japan.
E-mail: gkagiya@kitasato-u.ac.jp

Published by Elsevier Inc. on behalf of The American Society of Gene and Cell Therapy.
This is an open access article under the CC BY license (http://creativecommons.org/licenses/by/4.0/).

p53,[9] leading to the expression of pro-apoptotic proteins (e.g., Bax, Noxa, and Puma),[10] which promote the release of cytochrome *c* from mitochondria, which subsequently activates Apaf-1, caspase-9, and executioner caspases (caspase-3, -6, and -7).[11,12] This cascade ultimately leads to the activation of caspase-activated DNase, which induces apoptosis by fragmenting DNA.[13] Alternatively, in the cell membrane damage pathway, radiation activates acid sphingomyelinase located in caveolae (small invaginations in the cell membrane) resulting in increased ceramide production.[14] This cascade activates c-Jun N-terminal kinase, leading to apoptosis independently of p53.[15]

In vivo radiation-induced apoptosis in tumor cells is believed to occur in a similar manner to that *in vitro*. However, within the complex environment of a living organism, additional factors (e.g., tumor microenvironment, immune cell presence, and blood flow), which are absent *in vitro*, can significantly influence cellular responses. Consequently, the mechanisms underlying radiation-induced apoptosis observed *in vivo* may differ from those *in vitro*. Indeed, studies in animal models and humans have demonstrated that the extent of apoptosis following radiation therapy is often lower than expected and have pointed to differences in underlying molecular mechanisms.[16] Nevertheless, numerous studies have shown a correlation between the frequency of apoptosis within tumors and the efficacy of tumor shrinkage.[17] Understanding these mechanisms is crucial for developing new therapeutic strategies to enhance radiation therapy efficacy and represents an urgent area of research.

The oxygen tension within solid tumors is highly heterogeneous, and hypoxic regions are formed owing to an imbalance between cell proliferation and angiogenesis, in addition to temporary obstruction or reduced blood flow caused by abnormal vasculature. In response to hypoxic stress, tumor cells promote the expression of hypoxia-inducible factors (HIFs). HIFs are transcription factors composed of two subunits, HIFα and HIFβ. HIF-1α is rapidly degraded under normoxic conditions but stabilizes and translocates to the nucleus in hypoxic environments. It forms a heterodimer with HIF-1β, and the resulting transcriptional complex associates with histone acetyltransferases to bind to the hypoxia-responsive element (HRE) within the genome. This binding activates the expression of the target genes involved in angiogenesis, metabolic adaptation, acid tolerance, cell survival, and metastasis. As a result, the activation of HIF-1 allows tumor cells to survive and proliferate, even under hypoxic conditions. However, these hypoxic regions exhibit radioresistance, which presents a significant challenge in radiation therapy. Therefore, visualizing cell death under hypoxic conditions is a promising approach for assessing the efficacy of radiation therapy and optimizing treatment strategies.[18]

Real-time imaging of apoptosis is a crucial technique in cancer research, aiding in the evaluation of therapeutic efficacy and the elucidation of cell death mechanisms. Among these techniques, bioluminescence imaging systems have been developed to enable minimally invasive apoptosis detection. Notably, a system utilizing cyclic luciferase (luc) based on protein splicing (PS) has been reported as a method for visualizing apoptosis. PS is a post-translational process catalyzed by an intervening polypeptide segment known as an intein. Without the need for external energy, the intein facilitates a reaction that joins two flanking polypeptides, termed exteins, located at its ends, resulting in the excision of the intein and the formation of a shorter polypeptide.[19] When splicing occurs between two different polypeptides, it is referred to as protein *trans*-splicing (PTS). In PTS, two inteins (DnaEn and DnaEc) and their corresponding exteins from separate molecules interact, leading to joining of the exteins and the formation of a single, continuous polypeptide.[20] Using a PTS reaction, Kanno et al. developed a cyclic luc system in which two split-luc gene fragments were connected in inverse order with a DNA sequence encoding the DEVD peptide, a caspase-3 substrate.[21] The system also contained DNA fragments encoding DnaE inteins flanking the luc gene. The translated polypeptide undergoes circularization via the PTS activity of the DnaE inteins. Although circularized luc loses its enzymatic activity, cleavage of the DEVD motif by caspase-3 during apoptosis leads to linearization of luc, restoring its activity and enabling light emission via luciferin oxidation. Furthermore, the system designed to function specifically under hypoxic conditions using HRE is a valuable tool for screening anticancer drugs targeting tumors located in hypoxic regions.[18]

Here, we constructed a circular luc system using two types of luc and compared and evaluated its functionality for the minimally invasive, real-time detection of apoptosis, including that occurring in hypoxic tumor cells. Additionally, using the developed system, we visualized and quantified radiation-induced apoptosis within tumors and attempted to elucidate the mechanisms underlying radiation-induced apoptosis within the tumor microenvironment.

RESULTS

Evaluation of luminescent characteristics of an apoptosis imaging reporter using firefly and shrimp luciferases *in vitro*

Kanno et al. developed a gene encoding an artificial polypeptide that forms a circular structure post translation via PTS action, resulting in the loss of luc activity.[21] Upon apoptosis, activated caspase-3 cleaves the DEVD sequence, converting the structure to a linear form and restoring luc activity (Figure S1). This luc-containing polypeptide, which serves as an apoptosis reporter, was termed the apoptosis imaging reporter (AIR). To establish a sensitive apoptosis detection system, we used luc genes from fireflies and shrimp (*Oplophorus gracilirostris*) to construct plasmids cytomegalovirus (CMV)-cFluc and CMV-cNLuc, which regulate the expression of their respective AIRs under a CMV promoter (human CMV immediate-early enhancer and promoter, Figure S2). These plasmids were stably introduced into Chang, CHO AA8, and EM9 cells to generate four AIR-expressing cell lines: Chang/CMV-cFluc, Chang/CMV-cNLuc, CHOAA8/CMV-cFluc, and EM9/CMV-cFluc. Therefore, we investigated the luminescence properties during apoptosis *in vitro*. Following staurosporine (STS) treatment, which induces apoptosis, all cell lines exhibited increased luminescence. In Chang cells, shrimp-derived AIR showed ∼10-fold higher absolute luminescence

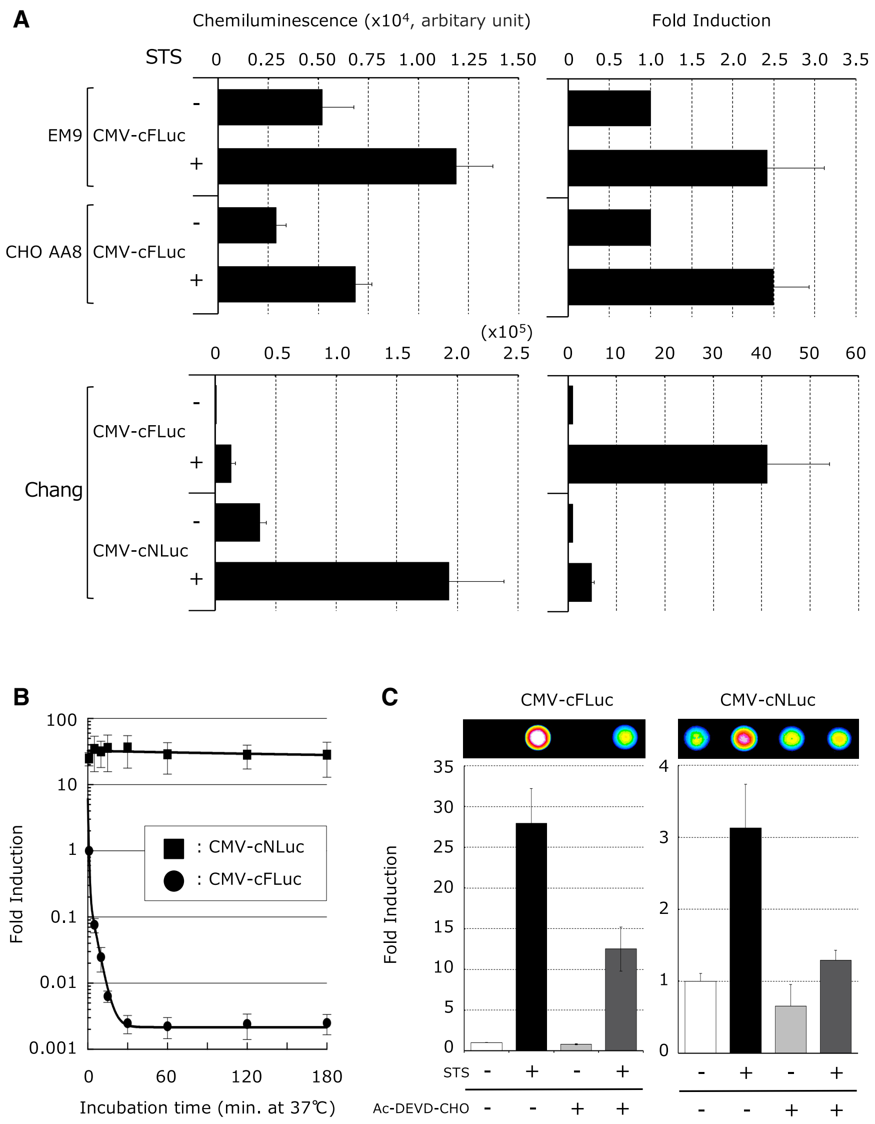

Figure 1. *In vitro* functional validation of AIR regulated by the CMV promoter
(A) The quantitative analysis of the luminescence generated by the luc activity of the AIR in response to STS treatment. Stable cell lines EM9/CMV-cFLuc, EM9/CMV-cNLuc, CHO-AA/CMV-cFLuc, CHO-AA/CMV-cNLuc, Chang/CMV-cFLuc, and Chang/CMV-cNLuc, which express firefly or shrimp-derived AIR under the CMV promoter, were treated with 1 μM STS for 3 h and then lysed in PLB before conducting luc assay. Each luminescence value was normalized against that of the cell lysate from the STS-untreated sample (*n* = 5 to 7). (B) Comparison of the thermal stability of luc activity between firefly- and shrimp-derived AIR. Cell lysates extracted from stable cell lines Chang/CMV-cFLuc and Chang/CMV-cNLuc were incubated at 37°C for up to 3 h. Fold induction of luminescence at different time points were calculated relative to the luminescence of Chang/CMV-cFLuc at 0 min (*n* = 5). (C) Suppression of luminescence by the caspase-3 inhibitor Ac-DEVD-CHO. Stable cell lines Chang/CMV-cFLuc and Chang/CMV-cNLuc were treated with 1 μM STS for 3 h after incubating the cells in medium containing 100 μM Ac-DEVD-CHO for 1 h. Luminescence was captured using an EMCCD camera. Fold inductions were calculated by dividing the luminescence of the harvested cell extracts treated with or without STS and with or without Ac-DEVD-CHO by that of cells without any treatment. Data are presented as the mean ± SD (*n* = 5).

relative to firefly AIR. In contrast, firefly-derived AIR showed a higher fold induction than shrimp-derived AIR (Figure 1A).

Next, we evaluated the thermal stability of the AIR. Using the initial luminescence after passive lysis buffer (PLB, Promega, Madison, WI, USA) treatment of Chang/CMV-cFluc (0 min) as a reference, Chang/CMV-cNLuc exhibited ∼25-fold higher luminescence. Even after 3 h at 37°C, Chang/CMV-cNLuc remained stable, whereas Chang/CMV-cFluc luminescence dropped sharply after 30 min, indicating a lower thermal stability (Figure 1B).

Additionally, to confirm that caspase-3-mediated DEVD cleavage drives an increase in luminescence, we evaluated luminescence changes upon the addition of the caspase-3 inhibitor Ac-DEVD-CHO. This significantly suppressed STS-induced luminescence in both Chang/CMV-cFluc and Chang/CMV-cNLuc cells, confirming that AIR detects apoptosis via luc reconstitution upon caspase-3 activation (Figure 1C).

To further evaluate the correlation between apoptosis and luminescence, we quantified TdT-mediated dUTP-biotin nick end labeling (TUNEL)-positive cells, caspase-3 activity, and luminescence in Chang/CMV-cFluc cells in response to changes in STS concentrations. The results showed that up to an STS concentration of 0.5 μM, the number of TUNEL-positive cells, caspase-3 activity, and luminescence increased linearly. However, at >0.5 μM, the rate of increase decreased (Figures 2A and 2B). Furthermore, the number of TUNEL-positive cells and luminescence, as well as caspase-3 activity and luminescence, was almost proportional, demonstrating that apoptosis can be estimated based on luminescence (Figures 2C and 2D).

Evaluation of *in vitro* luminescence characteristics of AIR regulated by a hypoxia-responsive promoter

We constructed 6HRE-cFLuc and 6HRE-cNLuc to regulate AIR using a hypoxia-responsive promoter (HRP) composed of six hypoxia-responsive elements (HREs) and a CMV IE1 core promoter (Figure S2). These plasmids were stably introduced into Chang cells

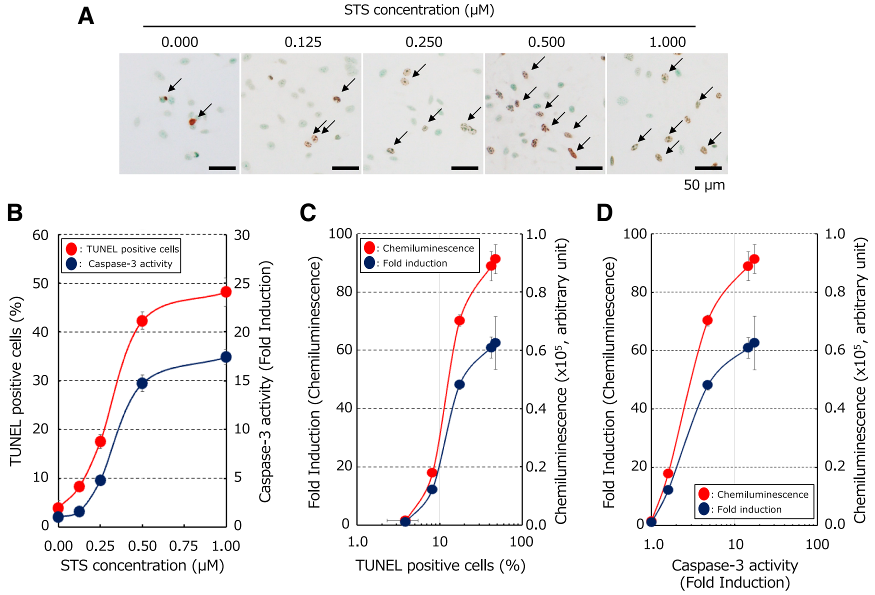

Figure 2. Changes in luminescence, TUNEL-positive cells, and caspase-3 activity with varying STS concentrations

(A) Detection of STS-induced apoptosis by TUNEL staining *in vitro*. Nuclei of the stable Chang/CMV-cFLuc cell line were stained with methyl green. TUNEL-positive cells exhibiting specific nuclear features (arrows) were identified as apoptotic. (B) Changes in TUNEL-positive cells and caspase-3 activity with varying STS concentrations. Fold induction was calculated based on the caspase-3 activity value of the untreated group. (C) The relationship between TUNEL-positive cell counts and luminescence values. Fold induction was calculated based on the number of TUNEL-positive cells in the untreated group. (D) The relationship between caspase-3 activity and luminescence values. Fold induction was calculated based on the caspase-3 activity value in the untreated STS-treated group.

to generate two AIR-expressing cell lines, Chang/6HRE-cFLuc and Chang/6HRE-cNLuc. Evaluation of the luminescence properties revealed that both exhibited the highest luminescence under hypoxic conditions (0.1%) with STS. Notably, Chang/6HRE-cNLuc showed 137-fold induction under 0.1% O_2 with STS, significantly surpassing the 17-fold induction of Chang/6HRE-cFLuc (Figure 3A). Additionally, both cell lines showed minimal luminescence at oxygen levels of >5%, confirming the detection of hypoxia-specific apoptosis (Figure 3B).

BLI of apoptosis induction in cells under normoxic and hypoxic conditions by various anticancer drugs

Using Chang/CMV-cFLuc, Chang/6HRE-cFLuc, Chang/CMV-cNLuc, and Chang/6HRE-cNLuc, which exhibited higher luminescence and fold induction, we investigated whether bioluminescence imaging (BLI) of apoptosis and hypoxic apoptosis induced *in vitro* by various anticancer drugs could be achieved. After STS treatment, shrimp-derived AIR emitted higher luminescence relative to firefly-derived AIR. Cells expressing AIR under the control of the CMV promoter showed apoptosis with luminescence under both normoxic and hypoxic conditions. In contrast, cells expressing AIR under HRP showed apoptosis only under hypoxic conditions after STS treatment (Figure 4A). Similar results were observed with other anticancer drugs, including cisplatin (CDDP), doxorubicin (DOX), mitomycin C (MMC), etoposide (EtoP), and camptothecin (CPT) (Figures 4B and 4C). The hypoxia-activated prodrugs tirapazamine (TPZ) and evofosfamide (TH302) were cytotoxic under hypoxic conditions, and enhanced luminescence was observed only under hypoxic conditions when TPZ or TH302 treatment was applied, confirming that apoptosis induced by each anticancer drug, despite the different mechanisms of cytotoxicity, can be visualized through luminescence under both normoxic and hypoxic conditions.

Evaluation of *in vivo* luminescence characteristics induced by STS treatment in tumors formed by constructed recombinant cells

To assess low invasiveness and real-time imaging of apoptosis and hypoxic apoptosis in tumors, recombinant cells were injected subcutaneously into nude mice. Three hours after intraperitoneal STS administration, luminescence was observed in tumors implanted with EM9/CMV-cFLuc, Chang/CMV-cFLuc, Chang/6HRE-cFLuc, and Chang/CMV-cNLuc cells (excluding Chang/6HRE-cNLuc), indicating their potential for *in vivo* apoptotic imaging (Figure 5A). Contrary to the *in vitro* results, Chang cells expressing firefly-derived AIR showed higher fold induction than shrimp-derived AIR *in vivo*. Notably, almost no luminescence was observed in the Chang/6HRE-cNLuc cells (Figure 5B). To confirm whether the increased luminescence in tumors following STS treatment was due to apoptosis, TUNEL and cleaved caspase-3 staining were performed. The frequency of TUNEL-positive and cleaved caspase-3-positive cells in STS-treated tumors was 4.8 ± 1.7 and 3.4 ± 1.3 times higher, respectively, than in untreated tumors, indicating a significant increase (Figures 5C, 5D, S3A, and S3B). The frequency at which apoptosis was induced was similar to that at which luminescence from the tumor was induced, suggesting that luminescence was due to apoptosis. These *in vivo* experiments demonstrated that the constructed AIR system is capable of real-time imaging of apoptosis induced in tumors and under hypoxic conditions in tumors.

Imaging of X-ray-induced apoptosis

We performed real-time imaging of X-ray-induced and hypoxic cell apoptosis using cells stably expressing firefly-derived AIR. *In vitro*, EM9/CMV-cFLuc cells exposed to X-rays (20 Gy) showed a luminescence peak 6 h post irradiation, confirming X-ray-induced apoptosis

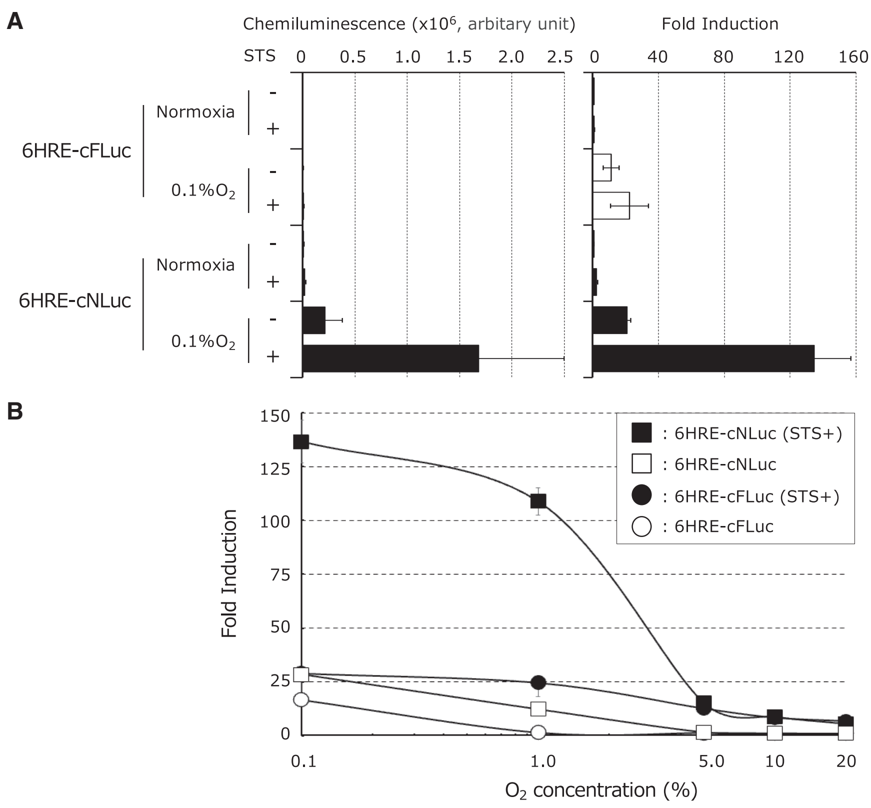

Figure 3. *In vitro* functional validation of AIR regulated by the HRP

(A) The quantitative analysis of the luminescence generated by the luc activity of the AIR in response to STS treatment under hypoxia. Stable cell lines Chang/6HRE-cFLuc and Chang/6HRE-cNLuc were preincubated for 12 h in 0.1% O_2, followed by treatment with 1 μM STS in 0.1% O_2 for 3 h. These cells were lysed in PLB before conducting luc assay. Luminescence values were expressed as fold induction, calculated by dividing the luminescence of extracts from treated cells by that of extracts from untreated cells under normoxia (n = 5 to 7). (B) The dependency of luminescence on oxygen concentration in hypoxic cell apoptosis. Stable cell lines Chang/6HRE-cFLuc or Chang/6HRE-cNLuc were preincubated for 12 h at different O_2 concentrations and then treated with 1 μM STS at corresponding oxygen concentrations for 3 h for luminescence measurements. The obtained luminescence values were expressed as the fold induction, calculated by dividing the luminescence of the harvested extracts from cells incubated at corresponding O_2 concentrations with or without STS by the luminescence of extracts at 20% O_2 without STS. Data are presented as the mean ± SD (n = 5).

(Figure 6A). In contrast, Chang/CMV-cFLuc cells showed decreased luminescence after irradiation (Figure 6B). Interestingly, *in vivo*, not only EM9/CMV-cFLuc but also Chang/CMV-cFLuc and Chang/6HRE-cFLuc (which did not show enhanced luminescence *in vitro*) exhibited increased luminescence after X-ray exposure, peaking at 9 h post irradiation—3 h later than *in vitro* (Figures 6C and 6D). Additionally, apoptosis was induced by X-rays in hypoxic regions; however, the fold induction of luminescence in these regions was lower than that in the oxygenated regions (Figure 6D).

Examination of increased luminescence involvement through the enhanced permeability and retention effect and CMV promoter activation by X-ray exposure

Owing to incomplete tumor vasculature, vascular permeability is increased, allowing drugs to leak into and spread within the tissue. Additionally, the immature lymphatic system in tumors is ineffective in clearing foreign substances, leading to the accumulation and retention of drugs leaking from the bloodstream within the tumor. This phenomenon, known as the enhanced permeability and retention (EPR) effect, increases in tumors, facilitating the accumulation of administered drugs. Based on the results showing that luminescence is suppressed by a caspase-3 inhibitor and that immunohistochemistry indicates an increase in the proportion of apoptotic cells, the increase in tumor luminescence was considered to be due to apoptosis. However, it remains possible that the enhanced EPR effect in the tumor caused the accumulation and retention of administered luciferin, leading to the increased substrate concentration and luminescence independent of the induction of apoptosis.

To verify the enhancement of luminescence through EPR mediated by radiation, the tumors were irradiated with X-rays, followed by luciferin injection to detect and image apoptosis. Imaging was performed before luciferin injection to assess the residual luminescence. Chang/FLuc cells expressing an intact luc gene with high luminescence were used to investigate the impact of residual luciferin. Luminescence from residual luciferin was detected only at 9 and 12 h post irradiation, likely due to the short intervals between luciferin administration. Additionally, the luminescence from luciferin accumulated via the EPR effect (luminescence ratio by EPR effect) was less than 15% of the luminescence detected immediately after the administration of luciferin, with a maximum detection rate of 11% ± 8.1% at 12 h post administration, irrespective of radiation exposure. This value was significantly lower than the 400% luminescence observed in tumors post irradiation and could only be detected with an exposure time 20 times longer than that of standard imaging after luciferin administration. These results indicated that the EPR effect was negligible in this *in vivo* experimental system (Figures 7A and 7B).

To evaluate CMV promoter activation within tumors due to X-ray irradiation, we used Chang/FLuc cells, which were also used to verify the EPR effect. Based on the luminescence ratio of irradiated to non-irradiated tumors immediately after X-ray exposure, the maximum

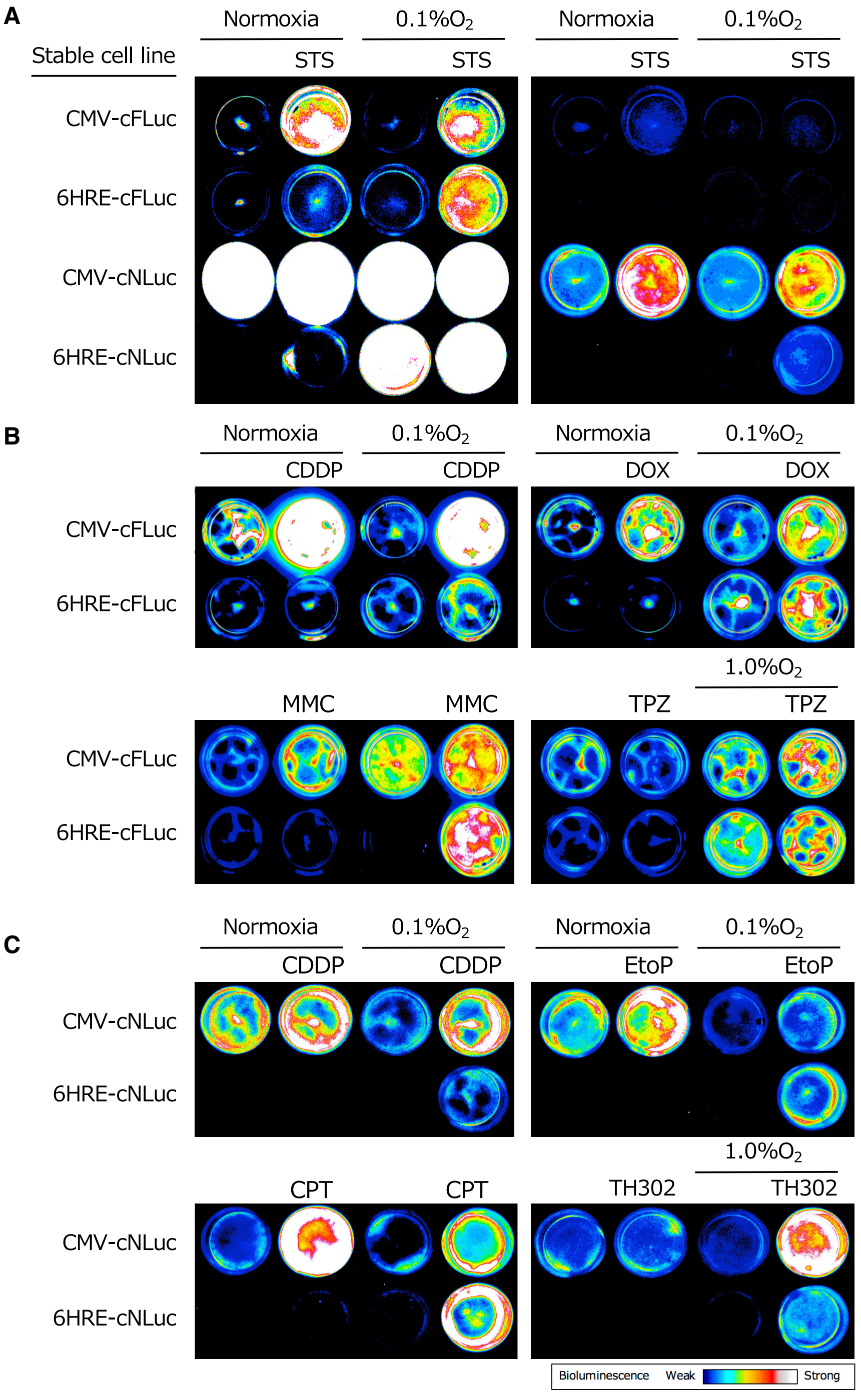

Figure 4. *In vitro* BLI of apoptosis and hypoxic cell apoptosis induced by various anticancer agents
Stable cell lines, Chang/CMV-cFLuc, Chang/6HRE-cFLuc, Chang/CMV-cNLuc, and Chang/6HRE-cNLuc, were incubated for 12 h under normoxia or 0.1% O_2 (1.0% O_2 in TPZ and TH302) and then treated under the same conditions with 1 μM STS for 3 h, 80 μM CDDP for 8 h, 8 μM DOX for 8 h, 60 μM MMC for 8 h, 20 μM TPZ for 24 h, 800 μM EtoP for 24 h, 20 μM CPT for 12 h, or 100 μM TH302 for 16 h. *In vitro* BLI was conducted after replacing culture medium with phenol red-free culture containing 500 μM D-luciferin or 50-fold diluted furimazine using an EMCCD camera mounted in a dark box. (A) *In vitro* BLI of apoptosis and hypoxic cell apoptosis induced by STS. The left shows images captured to detect luminescence from cells expressing firefly-derived AIR under control of the CMV promoter or HRP. In contrast, the right shows images of the same samples captured with a shorter exposure time to prevent signal saturation owing to the high luminescence of cells expressing shrimp-derived AIR. (B) *In vitro* BLI of apoptosis and hypoxic cell apoptosis induced by CDDP, DOX, MMC, and TPZ using cells expressing the firefly-derived AIR under the CMV promoter or HRP. (C) *In vitro* BLI of apoptosis and hypoxic cell apoptosis induced by CDDP, EtoP, CPT, and TH302 using cells expressing shrimp-derived AIR under the CMV promoter or HRP.

fold induction was 1.1 ± 0.13 at 6 h post irradiation, while the minimum fold induction was 0.8 ± 0.28 at 24 h post irradiation. No significant differences were observed between irradiated and non-irradiated groups. Thus, the activation of the CMV promoter by X-rays was minimal, and any effect was deemed negligible (Figure 7C). Therefore, it was concluded that both the EPR effect and CMV promoter activation due to X-rays were minimal and that the increase in luminescence induced by X-rays purely reflected apoptosis in the tumor.

Damage to tumor vasculature indirectly contributes to the induction of apoptosis within tumors through nutrient deprivation and hypoxia

Using the AIR system, we found that cells that did not undergo radiation-induced apoptosis *in vitro* underwent apoptosis after radiation exposure *in vivo* (Figures 6B and 6C). Recent studies have suggested that radiation upregulates antitumor immunity via type I interferons, promoting immune cell attacks against cancer cells. We hypothesized that *in vivo* apoptosis induced by radiation might be attributed to immune cell attacks due to radiation-induced immune activation. To test this, we used NOD/SCID mice, which lack T and B lymphocytes and have reduced natural killer (NK) cell activity. Tumors were established by subcutaneous implantation of Chang/CMV-cFLuc at two sites on the back of the mouse with X-ray irradiation on only one side. Even in immune-deficient mice, significant luminescence enhancement from the irradiated tumor was observed 9 h after 20 Gy irradiation, which was greater than that in nude mice, suggesting that the immune system was not involved in the induction of apoptosis early after irradiation

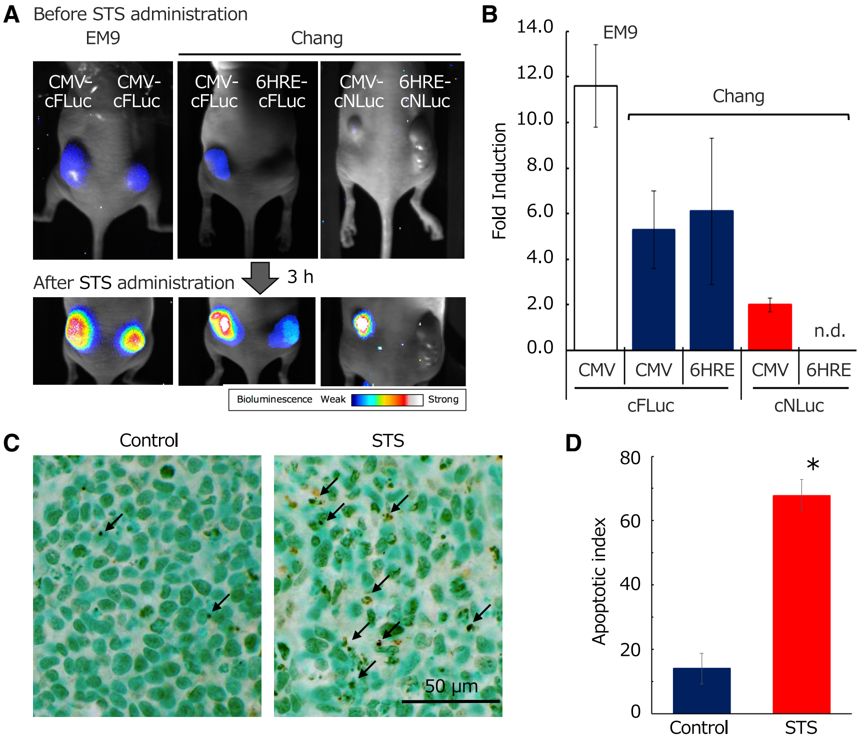

Figure 5. *In vivo* BLI of apoptosis and hypoxic cell apoptosis in tumors induced by STS and detection of apoptotic cells using TUNEL staining

(A) *In vivo* BLI of apoptosis and hypoxic cell apoptosis in tumors. Stable cell lines, EM9/CMV-cFLuc, Chang/CMV-cFLuc, Chang/6HRE-cFLuc, Chang/CMV-cNLuc, and Chang/6HRE-cNLuc, were subcutaneously transplanted into both sites of the backs of nude mice. When these tumors reached a diameter of 8–10 mm (approximately 2–3 weeks), an STS solution was intraperitoneally injected after the STS stock solution in DMSO was further dissolved in mineral oil. In *vivo* BLI was performed 3 h after the STS injection (100 μg/kg of mouse weight). (B) The quantitative analysis of the luminescence generated in tumors of the mice injected with STS. The detected luminescence values were expressed as the fold inductions calculated by dividing the luminescence value in the ROI of tumors from STS-treated mice by that of the ROI of tumors from STS-untreated mice (n = 11). (C) Detection of STS-induced apoptosis within tumors by TUNEL staining. Cell nuclei were stained with methyl green, and TUNEL-positive cells exhibiting specific nuclear features were identified as apoptotic cells. Arrows indicate apoptotic cells. (D) TUNEL-positive cells in STS-treated tumors vs. untreated tumors. The asterisk indicates a statistically significant difference (unpaired t test, p = 0.01). Data are presented as the mean ± SD (n = 4).

(Figure 8A). Indeed, immune cells, particularly lymphocytes and NK cells, do not infiltrate tumor tissue immediately after irradiation, and it has been reported that this process generally takes several days.[22,23]

We hypothesized that radiation-induced vascular damage contributes to apoptosis. To explore this, we evaluated the luminescence enhancement in tumors irradiated with 5 Gy, which caused less vascular damage. No significant increase in luminescence was observed in comparison to non-irradiated tumors (Figure 8B). Tumors irradiated with 20 Gy were examined for vascular morphology at 6 and 9 h post irradiation. Observations revealed extensive vascular damage, with fragmented capillaries particularly evident at 9 h post irradiation (Figure 8C). A blood flow velocity analysis showed that while non-irradiated tumors had flow velocities of approximately 40 μm/s, irradiated tumors had significantly reduced velocities of 20–10 μm/s at 6–9 h post irradiation (Figure 8D). These findings suggest that radiation damages the tumor vasculature, impairs blood flow, causes nutrient deprivation and hypoxia, and triggers apoptosis.

To examine the effect of nutrient deprivation on apoptosis, Chang/CMV-cFLuc cells used *in vivo* experiments were cultured in phosphate-buffered saline (PBS) instead of the culture medium to induce nutrient depletion, and luminescence changes were measured. No changes in luminescence were observed in the control group. However, in the group in which the medium was diluted 10-fold with PBS, luminescence increased 7.5-fold after 12 h, and in the group in which the medium was fully replaced with PBS, a 9.8-fold increase was observed after 4 h (Figure 8E). Using an apoptosis detection kit with labeled Annexin (Tali Apoptosis Kit), we found that 1.7% (1.7 ± 0.58) of control cells were apoptotic, while 8.7% (8.7 ± 0.58) of the PBS-replaced cells were apoptotic (Figure 8F). Caspase-3 activity also increased to 2.7 ± 0.4 in the PBS-replaced group in comparison to the control (Figure 8F), demonstrating that nutrient deprivation induces apoptosis in the Chang/CMV-cFLuc cells used for transplantation.

Moreover, severe tissue hypoxia can result from inadequate blood supply due to vascular injury. To investigate this, Chang/CMV-cFLuc cells were cultured under hypoxic conditions (1.0% and 0.1% oxygen) for 4 h, and the induction of apoptosis was assessed using luminescence from the luc assay. In comparison to normal culture conditions with 20% oxygen, fold induction of 2.4 ± 0.7 at 1% oxygen and 3.1 ± 0.8 at 0.1% oxygen was detected (Figure 8G). While X-ray irradiation of Chang/CMV-cFLuc cells did not induce significant apoptosis *in vitro*, apoptosis occurred *in vivo*, suggesting that radiation-induced tumor vasculature damage reduces nutrient (e.g., glucose and amino acids) and oxygen supply, in addition to direct apoptosis induction, altering the tumor microenvironment and indirectly triggering apoptosis.

DISCUSSION

In this study, we demonstrated that the AIR system enables accurate detection of apoptosis via caspase-3 activation. The caspase-3 inhibitor Ac-DEVD-CHO suppressed STS-induced luminescence, confirming its specificity. A correlation analysis among luminescence,

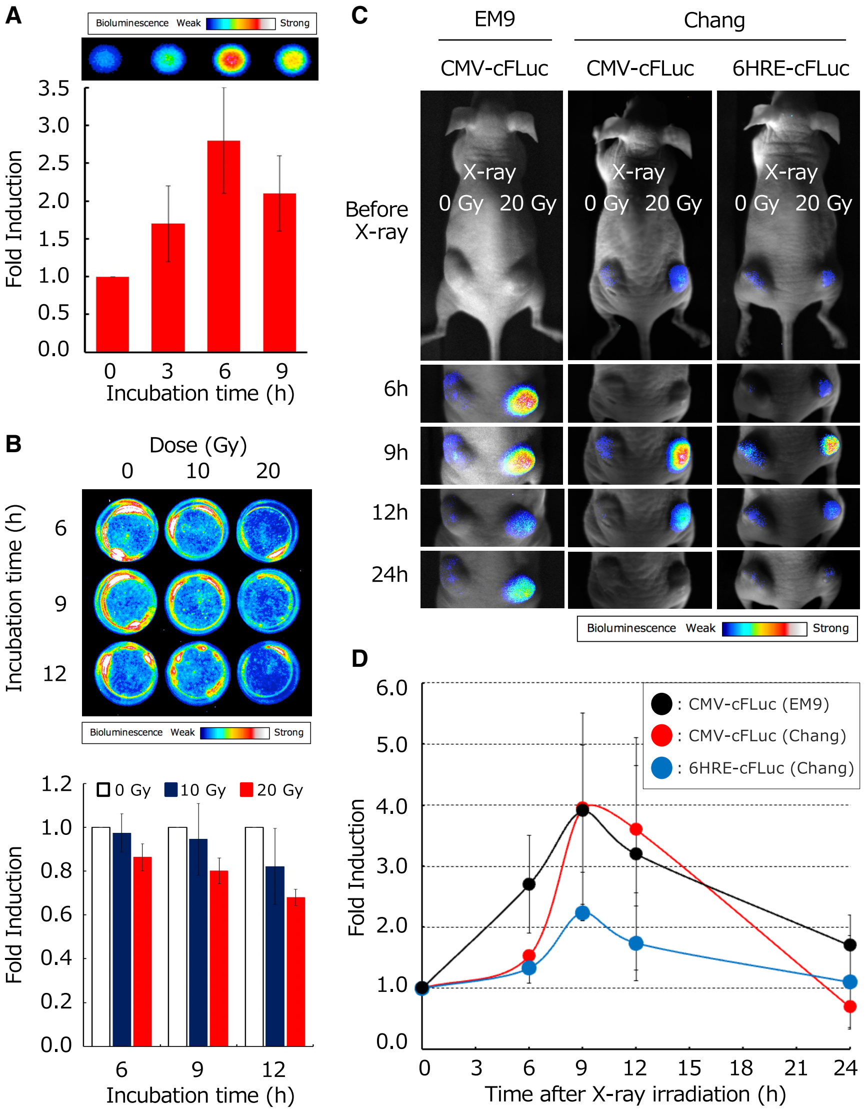

Figure 6. *In vitro* and *in vivo* BLI of apoptosis and hypoxic cell apoptosis induced by X-ray irradiation

(A) *In vitro* BLI and luminescence values from EM9/CMV-cFLuc cells after X-ray (20 Gy) irradiation. The luminescence values were expressed as the fold inductions calculated by dividing luminescence value obtained at each time point by luminescence value at 0 h (n = 4). (B) *In vitro* BLI and luminescence values from Chang/CMV-cFLuc cells 6, 9, and 12 h after X-ray irradiation at 0, 10, or 20 Gy. The luminescence values were expressed as the fold induction calculated by dividing a luminescence value obtained at each time point for each irradiation dose by the luminescence value at the corresponding time point for the 0 Gy dose (n = 3). (C) *In vivo* BLI of apoptosis and hypoxic cell apoptosis within tumors induced by X-ray. Stable cell lines, EM9/CMV-cFLuc, Chang/CMV-cFLuc, and Chang/6HRE-cFLuc cells, were subcutaneously transplanted into nude mice. When these tumors reached a diameter of 8–10 mm, they were irradiated with X-rays (20 Gy). BLI was performed before and 6, 9, 12, and 24 h after X-ray irradiation using an EMCCD camera. (D) Time courses of BLI intensity in tumors derived from stable cell lines EM9/CMV-cFLuc, Chang/CMV-cFLuc, and Chang/6HRE-cFLuc cells irradiated with X-rays (20 Gy). Data are presented as the mean ± SD (n = 8–10).

caspase-3 activity, and TUNEL-positive cells showed a linear luminescence increase up to 0.5 μM STS, indicating proportionality to caspase-3 activity. Above 0.5 μM, luminescence plateaued, suggesting D-luciferin depletion or signal saturation. Shrimp-derived AIR yielded stronger luminescence than firefly-derived AIR and maintained signal stability at 37°C, indicating superior thermal stability. This makes shrimp-derived AIR advantageous for long-term live-cell assays. These findings established AIR as a reliable quantitative reporter of caspase-3 activity, offering a robust platform for apoptosis detection.

We also demonstrated that the AIR system functions *in vivo*, as confirmed by experiments that induced apoptosis after anticancer drug administration. In an effort to enhance sensitivity, we tested shrimp-derived luc, which produced stronger signals *in vitro* than firefly-derived luc, which showed more robust luminescence *in vivo*. This difference in signal strength is likely due to the properties of furimazine, the substrate for shrimp-derived luc, which has low solubility in the blood and instability in biological environments,[24] leading to poor bioavailability and inefficient delivery to luc-expressing tumors. Indeed, while some luminescence was observed after intravenous administration *in vivo*, no luminescence was detected after intraperitoneal administration, which involves more steps before reaching the target tumor (data not shown). Recently developed derivatives of furimazine, hydrofurimazine, and fluorofurimazine exhibit improved solubility, enabling higher doses *in vivo* and significantly brighter bioluminescence signals than native furimazine.[24] Notably, fluorofurimazine provided the highest bioluminescence intensity, which was approximately 9-fold brighter than furimazine *in vivo*.[25] The use of these derivatives promises to enhance the sensitivity of our system further.

In our system, we examined radiation-induced apoptosis in Chang/CMV-cFLuc cells, which are currently capable of detecting apoptosis induced by anticancer agents with high sensitivity, using BLI in both *in vivo* and *in vitro* settings. Although we observed a significant enhancement in BLI *in vivo*, no induction of apoptosis was detected *in vitro*. In contrast, in EM9 cells, which have a mutation in XRCC1, an enzyme involved in single-strand DNA break repair, BLI enhancement was observed both *in vivo* and *in vitro*,

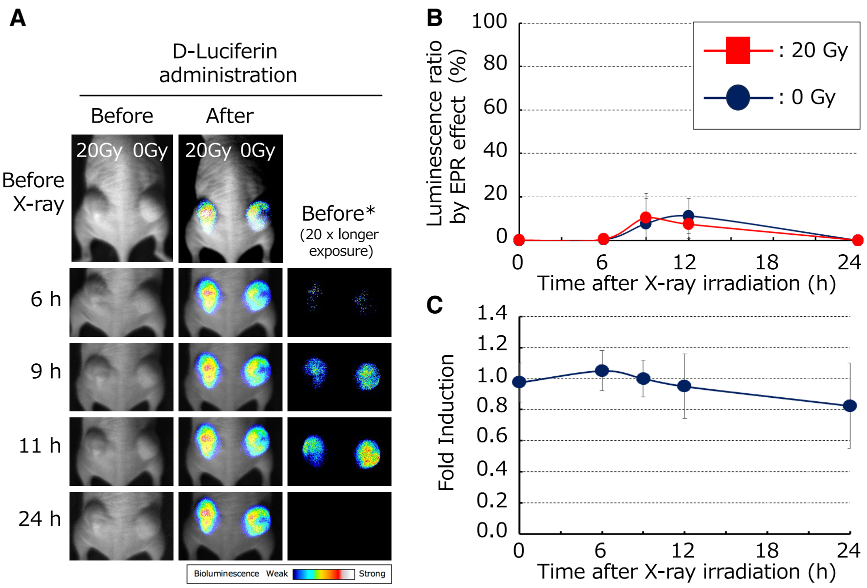

Figure 7. Verification of the enhancement of the EPR effect and CMV promoter activation by X-ray irradiation

(A) *In vivo* BLI by X-ray irradiation. Tumors transplanted with stable cell line Chang/pGL4.20 in the backs of nude mice were irradiated with X-rays (20 Gy). The term "Before*" refers to BLI conducted with 20-fold longer exposure time to visualize the weak luminescence prior to the administration of D-luciferin. (B) Verification of the enhancement of the EPR effect by X-ray irradiation. The luminescence ratio due to the EPR effect (%) was calculated by dividing the luminescence value in an ROI of the tumor before the administration of D-luciferin by the luminescence value in the same ROI after the administration of D-luciferin at each measurement time point. The data were then plotted (n = 3). (C) Verification of CMV promoter activation by X-ray irradiation. The RLU was calculated by dividing a luminescence value in an ROI of tumors irradiated with X-rays (20 Gy) by the luminescence value in the ROI of tumors not subjected to X-ray irradiation. Fold induction reflecting CMV promoter activity following irradiation was determined by dividing the RLU at each time point after X-ray irradiation by the RLU at 0 h. Data are presented as the mean ± SD (n = 3).

and the induction of apoptosis was confirmed *in vitro*. Thus, in the case of radiation-induced apoptosis in Chang/CMV-cFLuc cells, the enhancement of BLI observed only *in vivo* is thought to be associated with phenomena triggered exclusively *in vivo* by radiation exposure. Although the effects of EPR, indirect promoter activation, and immune involvement were ruled out, histological observations following irradiation revealed that the degree of vascular disruption was proportional to the radiation dose. This vascular damage leads to impaired nutrient and oxygen supplies. When these conditions were replicated *in vitro* without radiation exposure, apoptosis induction was confirmed. Furthermore, no apoptosis was induced in Chang cells with 5 Gy irradiation, where the degree of vascular damage was minimal. These findings suggest that direct radiation-induced apoptosis is rare in Chang cells, which exhibit low radiosensitivity. Instead, apoptosis observed *in vivo* appears to be indirectly mediated by nutrient and oxygen deprivation caused by radiation-induced damage to the tumor vasculature.

Previous studies have reported that radiation therapy may induce cancer cell death through indirect mechanisms. These reports suggest that the potential causes include the enhancement of cancer immunity, disruption of the tumor microenvironment, and induction of hypoxia or starvation resulting from damage to vascular endothelial cells. By constructing mathematical models, Rodríguez-Barbeito et al.[26] and Kawahara et al.[27] proposed that radiation exerts a profound indirect influence on cancer cell survival largely through vascular damage. In contrast, Song et al. demonstrated in animal models that vascular damage caused by radiation (15–20 Gy) leads to tumor tissue hypoxia, which indirectly contributes to cell death.[28] Castle and Kirsch showed that in mice with weakened endothelial cells due to Atm gene suppression, stereotactic body radiation therapy (SBRT) caused vascular damage, but the contribution of radiation to tumor regression was minimal.[29] However, in the present study, when radiation (20 Gy) was administered to tumors composed of radiation-sensitive EM9 cells and radiation-resistant Chang cells, while the apoptotic signals from Chang cells were relatively weak at first, they caught up at 9 h post irradiation and subsequently continued to show slightly stronger signals. This observation raises the possibility that, while apoptosis is not the sole determinant of cell death, under certain circumstances, the indirect effects of radiation may surpass its direct cytotoxic effects. Further investigations of this phenomenon are required.

The indirect effects of radiation (e.g., those affecting tumor vasculature) have long been implicated in cancer therapy through mathematical and animal models. In this study, we provide clear evidence that radiation-induced vascular damage, observed in real time through minimally invasive techniques, disrupts the nutrient and oxygen supply to tumor cells, thereby inducing apoptosis. These findings suggest a potential therapeutic approach for the treatment of cancer. However, these results were derived from a single cancer cell line model; thus, their generalizability is uncertain. Furthermore, this study focuses solely on apoptosis. While some studies have pointed out that radiation-induced apoptosis parallels radiotherapy outcomes in many cancers, nutrient deprivation and hypoxia resulting from tumor vascular damage are likely to induce not only apoptosis but also other forms of cell death (e.g., autophagic cell death and necrosis-like cell death). Our group has also developed cells that emit luminescence upon necrosis,[30] and we intend to pursue further studies using animal models to answer these questions. Moreover, we aimed to elucidate the extent to which vascular

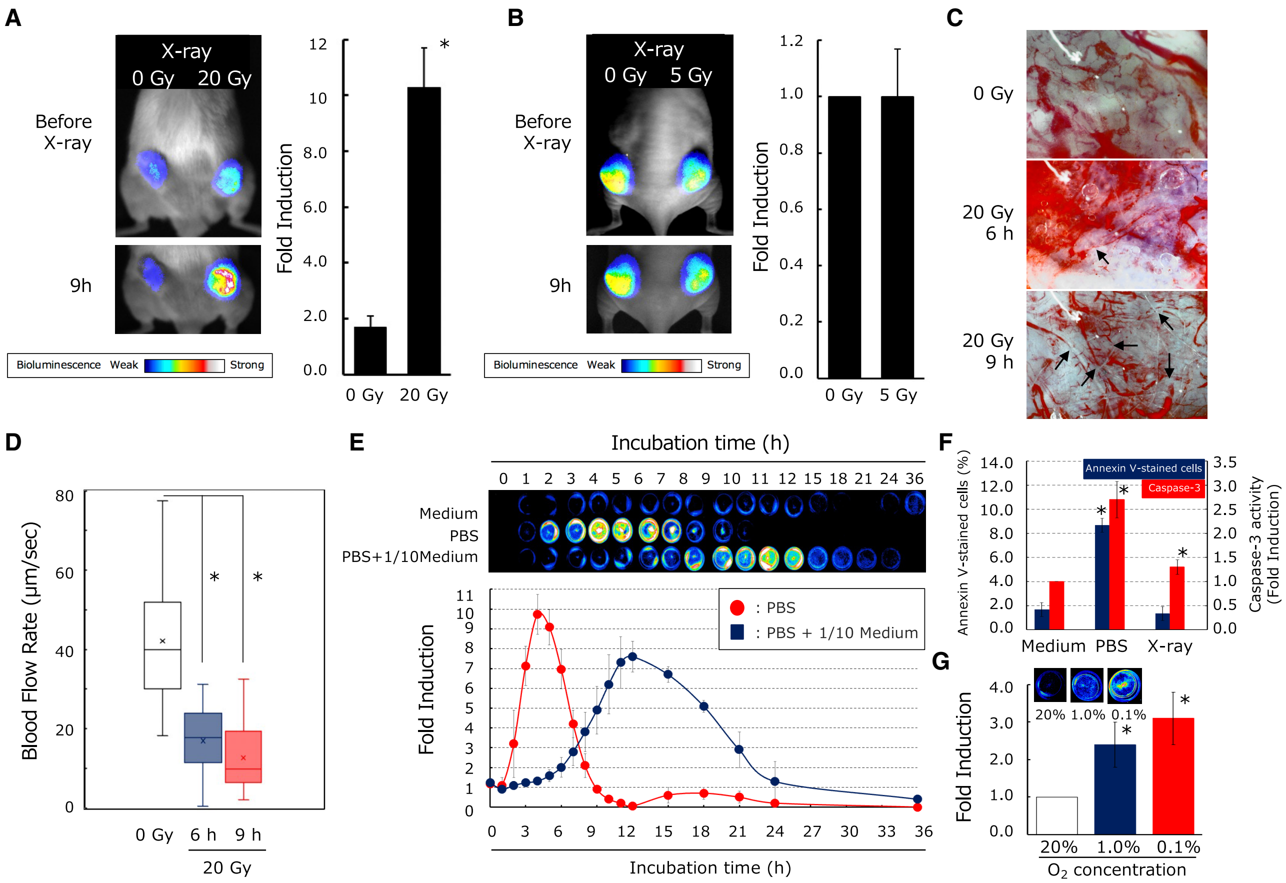

Figure 8. Damage to tumor vasculature indirectly contributes to the induction of apoptosis within tumors through nutrient deprivation and hypoxia

(A) The involvement of the immune system in X-ray-induced apoptosis within tumors. The stable cell line Chang/CMV-cFLuc was transplanted into NOD/scid mice. The tumors, which reached approximately 8–10 mm in diameter, were irradiated with X-rays (20 Gy). BLI was conducted at 9 h post irradiation. Fold induction, indicative of apoptosis, was calculated by dividing the luminescence value in the tumor ROI 9 h after X-ray irradiation by the luminescence value in the same ROI before irradiation. Error bars indicate standard deviations, and the asterisk indicates a statistically significant difference (unpaired t test, $p < 0.01$, $n = 9$). (B) *In vivo* BLI of apoptosis in tumors irradiated with X-ray doses unlikely to cause vascular damage. The stable cell line Chang/CMV-cFLuc was transplanted into nude mice. The tumors were irradiated with X-rays (5 Gy). BLI was conducted at 9 h post irradiation. Fold induction was calculated by dividing the luminescence ratio between the irradiated and non-irradiated tumors 9 h after X-ray irradiation by the luminescence ratio before irradiation. The error bars indicate standard deviations ($n = 3$). (C) Representative micrographs showing tumor vascular morphology in non-irradiated tumors and in tumors at 6 and 9 h after 20 Gy irradiation. Arrows indicate fragmented tumor vessels. (D) Comparison of blood flow velocities in tumor vessels between non-irradiated tumors and irradiated tumors at 6 and 9 h after 20 Gy irradiation ($n = 3$). The asterisk indicates a statistically significant difference (unpaired t test, $p < 0.01$, $n = 3$). (E) Induction of apoptosis by nutrient deprivation *in vitro*. The stable cell line Chang/CMV-cFLuc was cultured overnight, and then the culture medium was replaced with fresh medium, PBS, or medium diluted 10-fold with PBS. Following this replacement, PBS containing 500 μM D-luciferin was added at each time point for *in vitro* BLI. Fold induction was calculated by dividing the luminescence value at each measurement time by the luminescence value at 0 h after the culture medium change. Error bars indicate standard deviations ($n = 5$). (F) The analysis of apoptosis induction by nutrient depletion using fluorescein isothiocyanate (FITC)-labeled annexin V staining and a caspase-3 activity assay. Chang/CMV-cFLuc cells were subjected to nutrient depletion using PBS replacement or X-ray irradiation (20 Gy). After 9 h of incubation in the X-irradiated group and 4 h in the PBS-replaced group, cells were stained with FITC-labeled Annexin V, and apoptotic cells were counted. Additionally, caspase-3 activity was measured under the same conditions, and the induction rate was calculated based on the caspase-3 activity value of the untreated group. Asterisks denote statistically significant differences relative to the untreated group (unpaired t test, $p < 0.01$). Error bars represent standard deviation ($n = 3$). (G) The induction of apoptosis by hypoxia *in vitro*. The stable cell line Chang/CMV-cFLuc was cultured overnight and then incubated in 0.1% or 1% O_2 for 4 h. The medium was replaced with phenol red-free culture medium containing 500 μM D-luciferin, and the cells were subjected to *in vitro* BLI. Fold induction was calculated by dividing the luminescence value under hypoxia by the luminescence value under 20% O_2.

damage within tumor tissues influences therapeutic efficacy with greater precision. Our findings could fundamentally reshape the application of radiation for cancer treatment, leading to significant advancements in therapeutic efficacy.

MATERIALS AND METHODS

Reagents

All anticancer agents used in this study, including STS, CDDP, DOX, MMC, TPZ, TH302, EtoP, and CPT, were obtained from

Sigma-Aldrich, Inc. (MO, USA). These agents were dissolved in dimethyl sulfoxide (DMSO) or PBS and diluted to the desired concentrations in the culture medium. The caspase-3 inhibitor Ac-DEVD-CHO was purchased from Promega (Madison, WI, USA). Luminescent substrates, D-luciferin potassium salt for firefly luc and furimazine for shrimp luc, were obtained from Cayman Chemical (MI, USA) and Promega Corporation, respectively.

Cell culture

Chang liver cells (a HeLa contaminant), CHO AA8 cells (ovary-derived Chinese hamster), and EM9 cells (a single-strand break repair-deficient mutant derived from CHO AA8) were maintained in RPMI 1640 medium supplemented with 10% (v/v) heat-inactivated fetal calf serum, 100 U/mL penicillin, and 100 μg/mL streptomycin. All cells were cultured in a 5% CO_2 atmosphere at 37°C. For hypoxic treatments, cells were incubated in a 37°C hypoxic chamber with O_2 levels ranging from 0.1% to 10%, balanced with N_2.

Plasmid construction

To construct the plasmid CMV-cFLuc, which contains a cyclic luc gene flanked by DnaEn or DnaEc regions at both ends of a firefly luc gene fragment under the control of the CMV promoter, we used the plasmid pcFluc-DEVD developed by Kanno et al.[21] To clarify the notation of the constitutive sequence possessed by the plasmid (previously named pCMV),[18] the plasmid was designated as CMV-cFLuc in this study. The CMV promoter in CMV-cFLuc was replaced with an HRP containing 6 synthetic hypoxia-responsive elements (HREs) and the CMV IE1 core promoter,[18] resulting in plasmid 6HRE-cFLuc. For construction of the CMV-cNLuc plasmid, which expresses a shrimp-derived cyclic luc (cNLuc), the shrimp luc gene was split into an N-terminal fragment (158 amino acids) and a C-terminal fragment (11 amino acids). These segments were cloned in reverse order into pNL1.1 (Promega). A synthesized DNA fragment containing a caspase-3 recognition sequence, DEVD, was inserted between two fragmented shrimp luc segments. The two NLuc segments with the DNA sequence for DEVD were PCR amplified, and the firefly luc sequence between the DnaEc and DnaEn regions of CMV-cFLuc resulted in the plasmid CMV-cNLuc. To construct the plasmid p6HRE-cNLuc, which induces the expression of cNLuc under hypoxic conditions, a DNA fragment containing cNLuc was PCR amplified using CMV-cNLuc as a template and replaced with a DNA fragment containing cFLuc inserted between the EcoRI and BamHI sites in plasmid 6HRE-cFLuc. The orientation, integrity, and sequences of all plasmids were confirmed by nucleotide sequencing.

Transient transfection and establishment of stable cell lines

To assess the enhancement of luminescence via caspase-3-mediated restoration of luc activity, transient transfection was performed. Cells (5.0×10^5) were seeded into 30 mm glass Petri dishes and transfected 12 h later with CMV-cFLuc, CMV-cNLuc, 6HRE-cFLuc, or 6HRE-cNLuc plasmids using the Effectene transfection reagent (QIAGEN, CA, USA), in accordance with the manufacturer's instructions. After transfection, cells were incubated in fresh medium for 12 h before further experiments.

To establish stably transfected cell lines, 1.0×10^6 Chang liver, CHO AA8, or EM9 cells were seeded in 100 mm dishes and transfected the next day with plasmids carrying the neomycin/kanamycin gene (CMV-cFLuc, 6HRE-cFLuc, CMV-cNLuc, or 6HRE-cNLuc) or the hygromycin resistance gene (pGL4.50). Transfected cells were selected using 1 mg/mL G418 (Nacalai Tesque, Kyoto, Japan) or 200 μg/mL hygromycin B (FUJIFILM Wako, Osaka, Japan) for 14 days. Clones were selected, and luc gene integration was verified. The resulting stable cell lines were designated Chang/CMV-cFLuc, Chang/CMV-cNLuc, Chang/6HRE-cFLuc, Chang/6HRE-cNLuc, CHOAA8/CMV-cFLuc, CHOAA8/CMV-cNLuc, EM9/CMV-cFLuc, and EM9/CMV-cNLuc.

Luciferase reporter assay and *in vitro* BLI of apoptosis

Cells treated with anticancer agents were washed with PBS and lysed in 100 μL of PLB for 15 min at room temperature. Luc activity was measured using the Luciferase Assay System (Promega), according to the manufacturer's instructions. Luminescence fold induction was calculated by dividing the luminescence values of the treated samples by those of the controls. For *in vitro* BLI of apoptosis, cells were imaged with an EMCCD camera (iXon3 888, Andor Technology, UK) in phenol red-free medium containing 500 μM D-luciferin (FUJIFILM Wako, Osaka, Japan). For hypoxic conditions, cells were incubated for 12 h under 0.1%–10% O_2 before treatment. The cells were then re-incubated under hypoxic conditions after the addition of anticancer drugs or X-ray irradiation. For caspase-3 inhibition, cells were treated with 100 μM Ac-DEVD-CHO or 0.1% DMSO for 1 h, followed by STS treatment with Ac-DEVD-CHO for 3 h, prior to the luc assay.

Mouse xenograft tumor model

BALB/cA Jcl-nu/nu and NOD/ShiJic-scid Jcl female immunodeficient mice (5–7 weeks old) were purchased from CLEA Japan, Inc. (Tokyo, Japan). Stably transfected cells (5.0×10^6) in PBS mixed with Matrigel Matrix (Corning, NY, USA) were subcutaneously injected into both sides of the mouse back. Experiments were conducted 2–3 weeks post implantation when the tumors were 8–10 mm in diameter. All procedures were approved by the Animal Experiment Committee of Kitasato University (authorization numbers 23-02-1, 22-16-2, and 22-16-1) in compliance with national and international guidelines.

X-ray irradiation

Cells and tumors were irradiated using an MX-160 Labo X-ray system (mediXtec, Matsudo, Japan) at 160 kV and a dose rate of 2 Gy/min. The mice were anesthetized with isoflurane, and 3-mm-thick lead shielding was used to protect the surrounding normal tissues.

In vivo BLI of tumor apoptosis

In vivo BLI of tumor apoptosis was performed using an EMCCD camera after intraperitoneal injection of D-luciferin or furimazine. Signal intensity was quantified as photon counts within a region of

interest (ROI) over the tumor using the Andor Solis Imaging software (Andor Technology, UK). The fold induction of apoptosis by STS was calculated by dividing post-treatment photon counts by pre-treatment counts in the same mouse. Accurate dosing of luciferin into the peritoneal cavity of mice is challenging, and the rate of absorption from the mesentery can vary. To precisely measure the induction of apoptosis, the fold-induction of apoptosis within the tumor by X-ray irradiation was determined by calculating the relative luminescence value (RLU), which represents the ratio of photon counts in the ROI of the X-ray irradiated tumor to those in the ROI of the non-irradiated tumor. The fold induction of apoptosis following X-ray irradiation was calculated by dividing the RLU at each time point after irradiation by the RLU at 0 h. Pseudo-color images were processed using ImageJ (v.1.50i; National Institutes of Health, Bethesda, MD, USA).

TUNEL and cleaved caspase-3 staining, and quantification of caspase-3 activity

TUNEL-positive cells were detected using an *in situ* cell death detection kit (Roche). Tissue sections were deparaffinized, microwaved in 10 mM citrate buffer (pH 6.0) for 15 min, and then treated with TUNEL and methyl green (Muto Pure Chemicals Co. Ltd., Tokyo, Japan). Regarding cleaved caspase-3 staining, deparaffinization was also followed by microwave heating for 15 min. The tissue sections were then incubated overnight at 4°C with the primary antibody against cleaved caspase-3 (Cell Signaling Technology, Danvers, MA, USA, #9664). The secondary antibody was SimpleStain MAX-PO (Nichirei Biosciences, Inc, Tokyo, Japan). The number of TUNEL-positive cells and cleaved caspase-3-positive cells per high-power field (HPF) was quantified by counting TUNEL-positive and cleaved caspase-3-positive cells at 40 HPF. Caspase-3 activity was quantified using the QuantiFluo Caspase-3 Assay Kit (BioAssay Systems, CA, USA) using a fluorometric detection method.

Tumor vascular morphology and blood flow measurement

After irradiation, the skin covering the tumor in the mice was incised under anesthesia, and the morphology of the tumor vessels was directly observed using a capillaroscope (GOKO Bscan-ZD, GOKO Imaging Devices Co., Ltd., Tokyo, Japan). Blood flow velocity was quantified using GOKO-VIP (Velocity of Images and Pixels).

Apoptosis assay

After X-ray (20 Gy) irradiation or nutrient depletion by PBS, Chang/CMV-cFLuc cells were washed with PBS, suspended in binding buffer (10 mM HEPES, 140 mM NaCl, 2.5 mM $CaCl_2$, pH 7.4), and incubated with FITC-annexin V and propidium iodide for 10 min each in the dark. Apoptotic cells were quantified using a Tali Image Cytometer (Invitrogen).

Statistical analysis

All values are expressed as mean ± standard deviation. Significant differences between groups were determined using an unpaired t *test* or one-way functional ANOVA with Holm-Sidak's multiple comparison test.

DATA AVAILABILITY

All data presented in this work are available from the authors upon request.

ACKNOWLEDGMENTS

We thank Dr. Ozawa (the University of Tokyo) for sharing the DNA sequence information of a real-time sensing plasmid, pcFLuc-DEVD, for cas-3 activites. This research was supported in part by Grants-in-Aid for Scientific Research (C) (21K07654 and 24K10819) from Japan Society for the Promotion of Science and by grants from Kitasato University School of Allied Health Sciences (Grants-in-Aid for Research Project, nos. 2022-1004 and 2021-1009).

AUTHOR CONTRIBUTIONS

All authors contributed extensively to the work presented in this paper. G.K. and R.O. conceived this research concept. G.K., R.O., and F.H. contributed to the experimental design, data interpretation, and data analysis. N.A., A.Y., H.T., M.A., A.S., and K.Y. conducted the cell and animal experiments with the help of G.K., and M.H. and T.M. performed the preparation of tumor tissue section. G.K. and R.O. prepared the manuscript.

DECLARATION OF INTERESTS

The authors declare no competing interests.

SUPPLEMENTAL INFORMATION

Supplemental information can be found online at https://doi.org/10.1016/j.omton.2025.200997.

REFERENCES

1. Chaput, G., and Regnier, L. (2021). Radiotherapy: Clinical pearls for primary care. Can. Fam. Phys. *67*, 753–757. https://doi.org/10.46747/cfp.6710753.
2. Baskar, R., Dai, J., Wenlong, N., Yeo, R., and Yeoh, K.W. (2014). Biological response of cancer cells to radiation treatment. Front. Mol. Biosci. *1*, 24. https://doi.org/10.3389/fmolb.2014.00024.
3. Malouff, T.D., Combs, S.E., and Trifiletti, D.M. (2021). Editorial: Exploring the Potential of Particle Radiotherapy: Helium, Neutrons, Carbon, and Other Heavy Ions. Front. Oncol. *11*, 740974. https://doi.org/10.3389/fonc.2021.740974.
4. Sia, J., Szmyd, R., Hau, E., and Gee, H.E. (2020). Molecular Mechanisms of Radiation-Induced Cancer Cell Death: A Primer. Front. Cell Dev. Biol. *8*, 41. https://doi.org/10.3389/fcell.2020.00041.
5. Rodriguez-Ruiz, M.E., Vitale, I., Harrington, K.J., Melero, I., and Galluzzi, L. (2020). Immunological impact of cell death signaling driven by radiation on the tumor microenvironment. Nat. Immunol. *21*, 120–134. https://doi.org/10.1038/s41590-019-0561-4.
6. Linkous, A., Geng, L., Lyshchik, A., Hallahan, D.E., and Yazlovitskaya, E.M. (2009). Cytosolic phospholipase A2: targeting cancer through the tumor vasculature. Clin. Cancer Res. *15*, 1635–1644. https://doi.org/10.1158/1078-0432.CCR-08-1905.
7. Savarin, M., Znidar, K., Sersa, G., Komel, T., Cemazar, M., and Kamensek, U. (2023). Tumor Radiosensitization by Gene Electrotransfer-Mediated Double Targeting of Tumor Vasculature. Int. J. Mol. Sci. *24*, 2755. https://doi.org/10.3390/ijms24032755.
8. Held, K.D. (1997). Radiation-induced apoptosis and its relationship to loss of clonogenic survival. Apoptosis *2*, 265–282. https://doi.org/10.1023/A:1026485003280.
9. Helt, C.E., Cliby, W.A., Keng, P.C., Bambara, R.A., and O'Reilly, M.A. (2005). Ataxia telangiectasia mutated (ATM) and ATM and Rad3-related protein exhibit selective target specificities in response to different forms of DNA damage. J. Biol. Chem. *280*, 1186–1192. https://doi.org/10.1074/jbc.M410873200.
10. Hao, Q., Chen, J., Lu, H., and Zhou, X. (2023). The ARTS of p53-dependent mitochondrial apoptosis. J. Mol. Cell Biol. *14*, mjac074. https://doi.org/10.1093/jmcb/mjac074.

11. Shimizu, S., Narita, M., and Tsujimoto, Y. (1999). Bcl-2 family proteins regulate the release of apoptogenic cytochrome c by the mitochondrial channel VDAC. Nature *399*, 483–487. https://doi.org/10.1038/20959.

12. Cheng, E.H., Wei, M.C., Weiler, S., Flavell, R.A., Mak, T.W., Lindsten, T., and Korsmeyer, S.J. (2001). BCL-2, BCL-X(L) sequester BH3 domain-only molecules preventing BAX- and BAK-mediated mitochondrial apoptosis. Mol. Cell *8*, 705–711. https://doi.org/10.1016/s1097-2765(01)00320-3.

13. Enari, M., Sakahira, H., Yokoyama, H., Okawa, K., Iwamatsu, A., and Nagata, S. (1998). A caspase-activated DNase that degrades DNA during apoptosis, and its inhibitor ICAD. Nature *391*, 43–50. https://doi.org/10.1038/34112.

14. Santana, P., Peña, L.A., Haimovitz-Friedman, A., Martin, S., Green, D., McLoughlin, M., Cordon-Cardo, C., Schuchman, E.H., Fuks, Z., and Kolesnick, R. (1996). Acid sphingomyelinase-deficient human lymphoblasts and mice are defective in radiation-induced apoptosis. Cell *86*, 189–199. https://doi.org/10.1016/s0092-8674(00)80091-4.

15. Pena, L.A., Fuks, Z., and Kolesnick, R. (1997). Stress-induced apoptosis and the sphingomyelin pathway. Biochem. Pharmacol. *53*, 615–621. https://doi.org/10.1016/s0006-2952(96)00834-9.

16. Fulda, S. (2009). Tumor resistance to apoptosis. Int. J. Cancer *124*, 511–515. https://doi.org/10.1002/ijc.24064.

17. Carneiro, B.A., and El-Deiry, W.S. (2020). Targeting apoptosis in cancer therapy. Nat. Rev. Clin. Oncol. *17*, 395–417. https://doi.org/10.1038/s41571-020-0341-y.

18. Kagiya, G., Ogawa, R., Hyodo, F., Yamashita, K., Nakamura, M., Ishii, A., Sejimo, Y., Tominaga, S., Murata, M., Tanaka, Y., and Hatashita, M. (2016). Development of a real-time imaging system for hypoxic cell apoptosis. Mol. Ther. Methods Clin. Dev. *5*, 16009. https://doi.org/10.1038/mtm.2016.9.

19. Southworth, M.W., Adam, E., Panne, D., Byer, R., Kautz, R., and Perler, F.B. (1998). Control of protein splicing by intein fragment reassembly. EMBO J. *17*, 918–926. https://doi.org/10.1093/emboj/17.4.918.

20. Mills, K.V., Lew, B.M., Jiang, S., and Paulus, H. (1998). Protein splicing in trans by purified N- and C-terminal fragments of the Mycobacterium tuberculosis RecA intein. Proc. Natl. Acad. Sci. USA *95*, 3543–3548. https://doi.org/10.1073/pnas.95.7.3543.

21. Kanno, A., Yamanaka, Y., Hirano, H., Umezawa, Y., and Ozawa, T. (2007). Cyclic luciferase for real-time sensing of caspase-3 activities in living mammals. Angew. Chem. Int. Ed. Engl. *46*, 7595–7599. https://doi.org/10.1002/anie.200700538.

22. Walle, T., Kraske, J.A., Liao, B., Lenoir, B., Timke, C., von Bohlen Und Halbach, E., Tran, F., Griebel, P., Albrecht, D., Ahmed, A., et al. (2022). Radiotherapy orchestrates natural killer cell dependent antitumor immune responses through CXCL8. Sci. Adv. *8*, eabh4050. https://doi.org/10.1126/sciadv.abh4050.

23. Takeshima, T., Chamoto, K., Wakita, D., Ohkuri, T., Togashi, Y., Shirato, H., Kitamura, H., and Nishimura, T. (2010). Local radiation therapy inhibits tumor growth through the generation of tumor-specific CTL: its potentiation by combination with Th1 cell therapy. Cancer Res. *70*, 2697–2706. https://doi.org/10.1158/0008-5472.CAN-09-2982.

24. Su, Y., Walker, J.R., Park, Y., Smith, T.P., Liu, L.X., Hall, M.P., Labanieh, L., Hurst, R., Wang, D.C., Encell, L.P., et al. (2020). Novel NanoLuc substrates enable bright two-population bioluminescence imaging in animals. Nat. Methods *17*, 852–860. https://doi.org/10.1038/s41592-020-0889-6.

25. Gaspar, N., Walker, J.R., Zambito, G., Marella-Panth, K., Lowik, C., Kirkland, T.A., and Mezzanotte, L. (2021). Evaluation of NanoLuc substrates for bioluminescence imaging of transferred cells in mice. J. Photochem. Photobiol., B *216*, 112128. https://doi.org/10.1016/j.jphotobiol.2021.112128.

26. Rodriguez-Barbeito, P., Diaz-Botana, P., Gago-Arias, A., Feijoo, M., Neira, S., Guiu-Souto, J., Lopez-Pouso, O., Gomez-Caamano, A., and Pardo-Montero, J. (2019). A Model of Indirect Cell Death Caused by Tumor Vascular Damage after High-Dose Radiotherapy. Cancer Res. *79*, 6044–6053. https://doi.org/10.1158/0008-5472.CAN-19-0181.

27. Kawahara, D., Nagata, Y., and Watanabe, Y. (2021). Improved cellular automata model shows that indirect apoptotic cell death due to vascular damage enhances the local control of tumors by single fraction high-dose irradiation. Biomed. Phys. Eng. Express *8*. https://doi.org/10.1088/2057-1976/ac4466.

28. Song, C.W., Lee, Y.J., Griffin, R.J., Park, I., Koonce, N.A., Hui, S., Kim, M.S., Dusenbery, K.E., Sperduto, P.W., and Cho, L.C. (2015). Indirect Tumor Cell Death After High-Dose Hypofractionated Irradiation: Implications for Stereotactic Body Radiation Therapy and Stereotactic Radiation Surgery. Int. J. Radiat. Oncol. Biol. Phys. *93*, 166–172. https://doi.org/10.1016/j.ijrobp.2015.05.016.

29. Castle, K.D., and Kirsch, D.G. (2019). Establishing the Impact of Vascular Damage on Tumor Response to High-Dose Radiation Therapy. Cancer Res. *79*, 5685–5692. https://doi.org/10.1158/0008-5472.CAN-19-1323.

30. Kagiya, G., Sato, A., Ogawa, R., Hatashita, M., Kato, M., Kubo, M., Kojima, F., Kawakami, F., Nishimura, Y., Abe, N., and Hyodo, F. (2021). Real-time visualization of intratumoral necrosis using split-luciferase reconstitution by protein trans-splicing. Mol. Ther. Oncolytics *20*, 48–58. https://doi.org/10.1016/j.omto.2020.12.001.

OMTON, Volume *33*

Supplemental information

Real-time imaging reveals radiation-induced intratumor apoptosis via nutrient and oxygen deprivation following vascular damage

Go Kagiya, Ryohei Ogawa, Toshihide Matsumoto, Fuminori Hyodo, Nanako Abe, Ami Yuzawa, Haru Takeuchi, Miki Aoyagi, Ayaka Sato, Kei Yamashita, and Masanori Hatashita

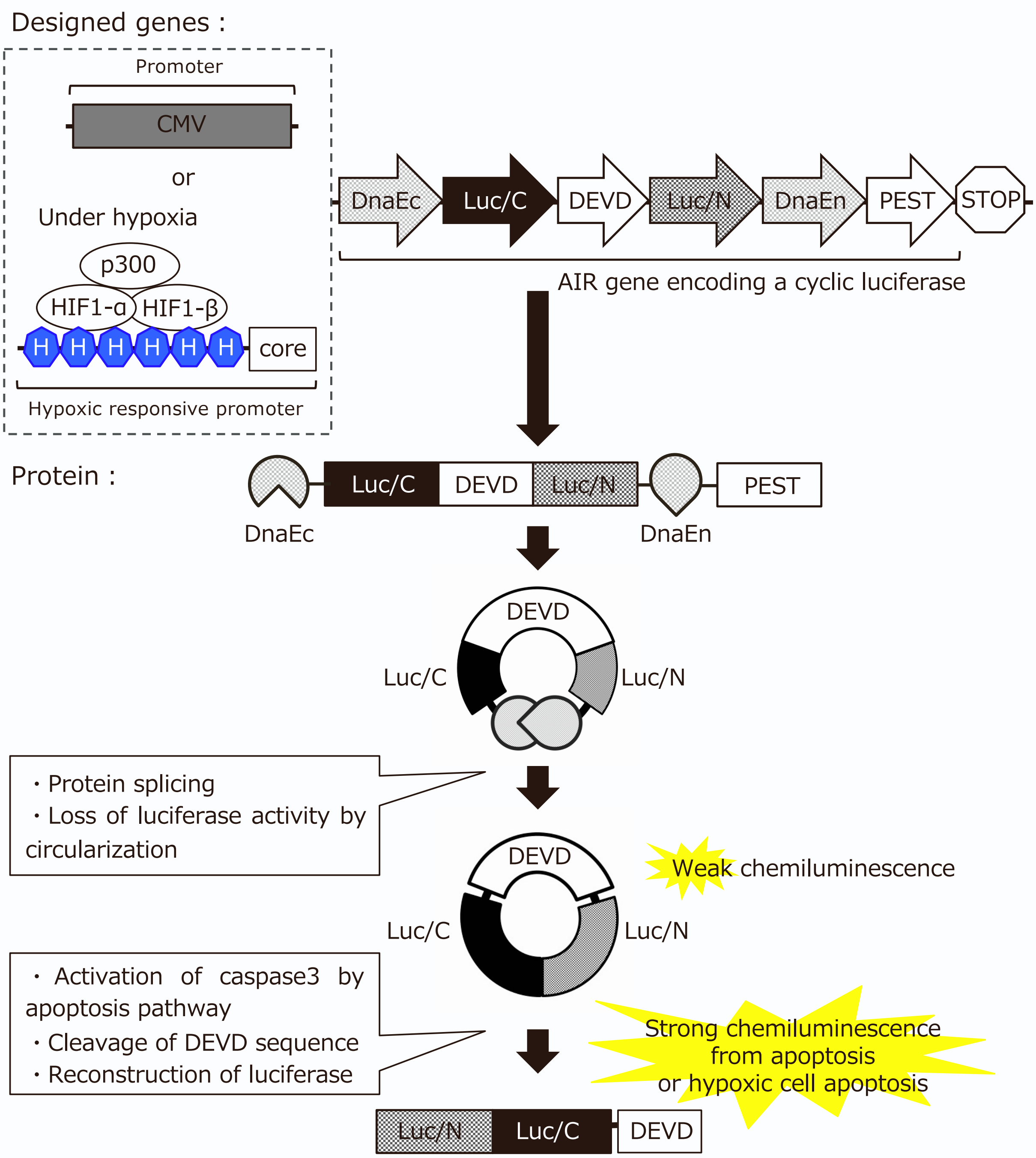

Figure S1. Schematic illustration for the detection of apoptosis and hypoxic cell apoptosis using apoptosis imaging reporter (AIR). In the plasmid used for detection of apoptosis, the expression of AIR is regulated by the CMV promoter. In contrast, for the detection of hypoxic cell apoptosis, a hypoxia-responsive promoter (HRP) that contains six hypoxia-responsive element and CMV IE1 core promoter, to which a heterodimeric complex composed of HIF-1α and HIF-1β binds, regulates the expression of AIR. Upon translation, the DnaEc and DnaEn inteins interact with each other to catalyze protein splicing, resulting in polypeptide circularization. If this event fails, rapid degradation occurs due to the PEST domain added at the N-terminus, which act as a signal peptide for protein degradation. The enzymatic activity of luc is lost when AIR distorted by circularization. However, the enzymatic activity is restored when the structure of luc becomes intact after caspase-3 cleaves the DEVD peptide in AIR during apoptosis. This results in high luminescence generation by luciferin oxidation.
CMV, human cytomegalovirus immediate early enhancer and promoter; H, hypoxia-responsive element; core, CMV IE1 core promoter; DnaEn, N-terminal fragment of DnaE intein; DnaEc, C-terminal fragment of DnaE intein; Luc/N, N-terminal fragment of luc; Luc/C, C-terminal fragment of luc; PEST, proteolytic signal to target proteins for degradation.

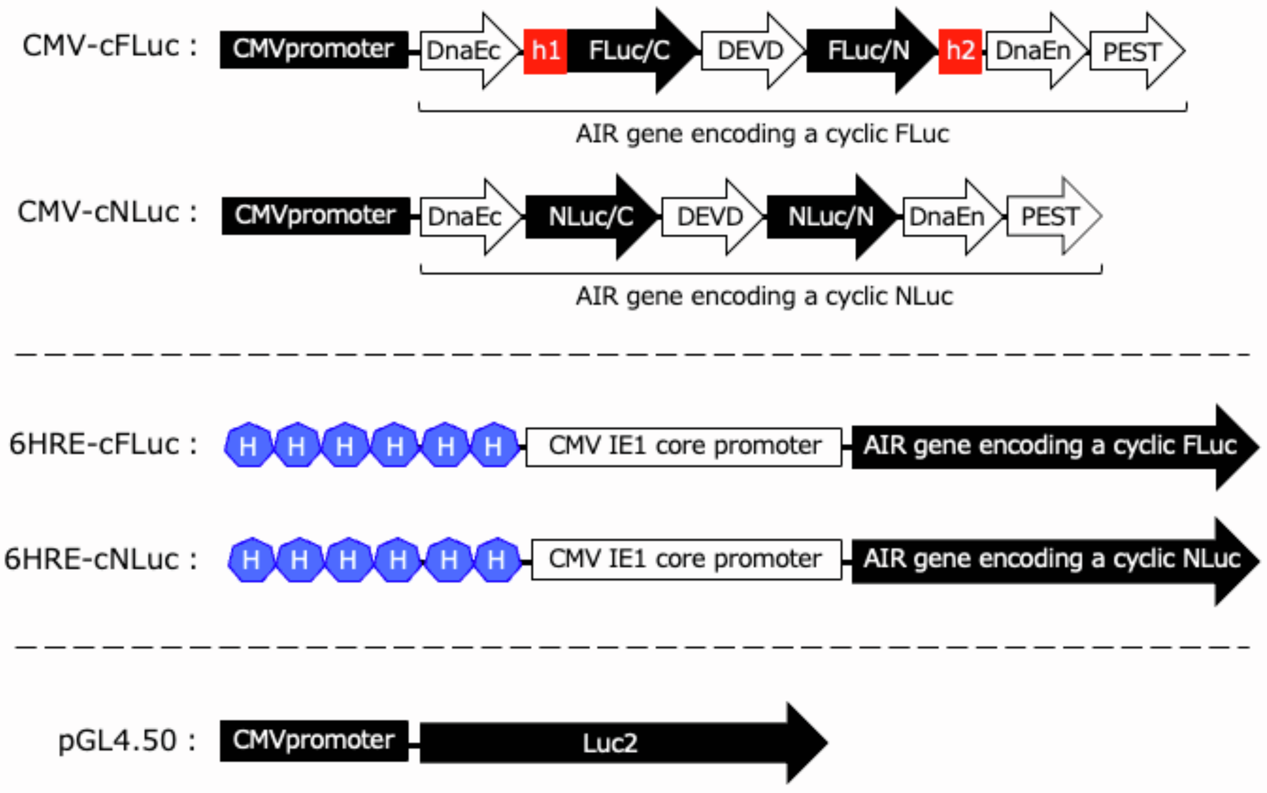

Figure S2. Schematic illustrations of the structures of the plasmids CMV-cFLuc, CMV-cNLuc, 6HRE-cFLuc, 6HRE-cNLuc, and pGL4.50 used in this study. The 'h1' and 'h2' in the top row represent two different hinge-array sequences. h1: CFNIS (one-letter amino acid sequence) and h2: KFAEYC (one-letter amino acid sequence). CMV promoter: CMV immediate early enhancer/promoter. The 'H' in the heptagon in the middle row denote the hypoxia-responsive element. H: cacgta (nucleotide sequence), Luc2: firefly luc gene.

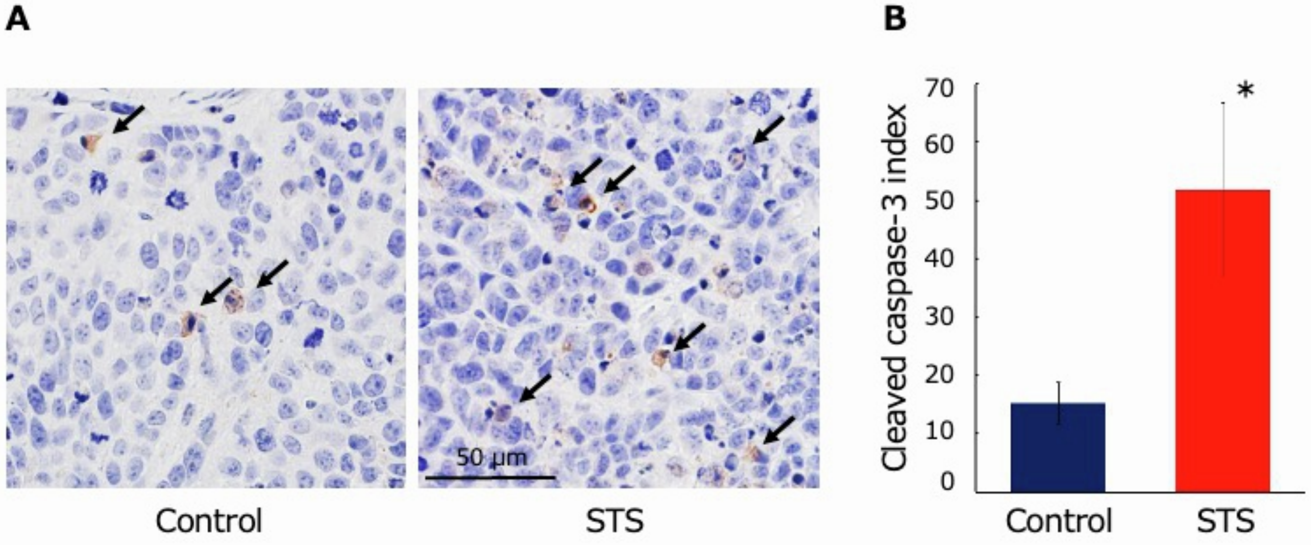

Figure S3. *In vivo* detection of STS-induced apoptotic cells using cleaved caspase-3 staining. A, Detection of cleaved caspase-3–positive cells in tumors. Cell nuclei were stained with Mayer's hematoxylin and cleaved caspase-3 positive cells (arrows) were identified as apoptotic cells. **B,** Cleaved caspase-3 positive cells in STS-treated tumors vs. untreated tumors. Asterisks indicate a statistically significant difference (unpaired t-test, $p = 0.01$). Data are presented as the mean ± SD (n = 4).
